# Trends in both Alzheimer’s disease and Diabetes mellitus related mortality among middle-aged and older adults in the United States, 1999 to 2023: a CDC WONDER database analysis

**DOI:** 10.1186/s12883-026-05058-2

**Published:** 2026-06-12

**Authors:** Aiman Javed, Shiva Kumar, Manahil Iftekhar, Hafiza Tooba Siddiqui, Ayesha Khan, Fatima Faisal, Muaz Ali, Fatima Sohail, Muhammad Hanif Khan

**Affiliations:** 1 Dow University of Health Sciences, Karachi, Pakistan; 2 People’s University of Medical and Health Sciences for Women (PUMHSW), Nawabshah, Sindh Pakistan; 3 Jinnah Sindh Medical University, Karachi, Sindh Pakistan; 4 Department of Neurology, Cleveland Clinic, Weston, FL USA; 5 Spinghar University, Kart-e-Naw, Sarak-e-Naw Rehman Mena 60m Street, Kabul, Afghanistan

**Keywords:** Alzheimer’s disease, Diabetes mellitus, Mortality trends, CDC WONDER database, Age-adjusted mortality rate.

## Abstract

**Objective:**

To analyze the temporal trends of both Alzheimer’s disease and Diabetes mellitus-related mortality in adults aged > 45years in the United States between 1999 and 2023, and to evaluate the changes in mortality patterns over time.

**Background:**

Alzheimer’s Disease and Diabetes Mellitus are two different diseases that have diverse underlying pathophysiology, but they often coexist, having common pathways. There is a high prevalence of concurrence between these two conditions, yet their combined mortality trend is underexplored.

**Methods:**

We utilize mortality data from the CDC Wide-Ranging Online Data for Epidemiologic Research (WONDER). Individuals aged > 45 were included who had both Alzheimer’s disease (G30) and Diabetes mellitus(E10-14). Age-adjusted mortality rates (AAMRs) and crude mortality rates (CMRs) per 100,000 were calculated and were standardized to the 2000 U.S population. Joint point regression models were used to identify the temporal variations and to calculate Annual Percentage Change (APC) and Average Annual Percentage Change (AAPC) with 95% confidence intervals.

**Results:**

Overall, a total of 224,082 deaths occurred in patients of both Alzheimer’s disease and Diabetes mellitus, in the age group ≥ 45 years, from 1999 to 2023. There is an upward trajectory noted from 2.82 in 1999 to 4.42 in 2023, with the highest incidence between 2017 and 2020, followed by a decline. Mortality rose in both sexes, with a persistently higher rate in females. The mortality rise from 1999 to 2023 in middle-aged people (45–64 years), and there was a rise in the trend of around 41% among adults ≥ 65 years. White individuals show higher deaths (78.9%), yet higher AAMR is observed in Black and Hispanic populations, showing racial disparities. Regionally, the West shows the highest AAMR, while non-metropolitan areas show higher mortality than metropolitan areas.

**Conclusion:**

The trend of mortality in individuals with both Alzheimer’s disease and Diabetes Mellitus has increased in the past two decades, but there is a sharp rise observed after 2020 that may show the impact of the COVID-19 pandemic. These findings emphasized public health strategies.

**Supplementary Information:**

The online version contains supplementary material available at https://doi.org/10.1186/s12883-026-05058-2.

## Introduction

Alzheimer’s disease (AD) is a progressive neurodegenerative disorder that results in memory problems, cognitive dysfunction, and loss of the ability to perform everyday activities. There is no cure for the disease, which primarily strikes those who are adults aged 65 years and older. Alzheimer’s disease AD resulted in more than 119,000 deaths in the United States, including being the seventh leading cause of death in America [1]. An aging population and greater coding of AD as a cause of death-on-death certificates explain the continued rise in AD deaths since 1999 [2, 3]. Overall and by sex, race, and state of residence area [4], age-adjusted rates were calculated per 100,000 population; increases in rates from 1999 to 2022 are presented. Diabetes mellitus, type 1 (T1DM) and type 2 (T2DM), are chronic metabolic disorders accompanied by prolonged elevated blood glucose, as well as consisting of prevalent microvascular and macrovascular complications. An estimated 38 million Americans, or one in nine adults, have diabetes [5]. Despite improved management and increased awareness, diabetes remains an important contributor to CVD mortality and premature mortality overall [6]. Diabetes mellitus was the eighth leading underlying cause of death in the United States in 2023, accounting for more than 95,000 death certificates [7]. Previous analyses show that, although DM mortality decreased significantly during the early 2000s, this trend may have stabilized or reversed in later years [8, 9].

The relationship between DM and AD can be localized more by the way that both have some common pathophysiologic mechanisms, including insulin resistance, vascular damage in the body, and inflammation (before they develop) [10]. Patients with diabetes are in much greater danger of developing AD and cognitive impairment, which is why these conditions must be addressed together in mortality surveillance [11]. Recent epidemiological studies suggested that DM increases the risk of dementia-related mortality if develops in midlife and persists for longer duration [12]. Time trends in AD and DM mortality over periods of time, where a common analytic design allowed for direct comparison, have rarely been compared. The CDC WONDER Multiple Cause of Death file for 1999–2023 presents age-adjusted, population-based rates by demographic characteristic. This study aims to quantify mortality trend involving both AD and DM among U.S. adults from ≥ 45 years older, with the objective of identifying temporal patterns, associations, and disparities to inform future prevention strategies, healthcare planning, and public health policy efforts in the United States.

## Methodology

### Study setting and population

This study utilizes data from the Centers for Disease Control and Prevention’s (CDC) Wide-Ranging Online Data for Epidemiologic Research (WONDER) website to examine trends in mortality related to coexisting Alzheimer’s disease and diabetes mellitus in all U.S. states from 1999 to 2023 [13]. The information was taken from the Multiple Cause of Death (MCOD) dataset, which aggregates data from U.S. resident death certificates.

Decedents aged more than 45 years were included if they had both Alzheimer’s disease and diabetes mellitus. The International Classification of Diseases, 10th revision (ICD-10) codes are G30 for Alzheimer’s disease and E10-14 for diabetes mellitus [14, 15]. These codes have been validated and used in prior studies.

This study utilizes publicly available, de-identified data from CDC WONDER database and has no human interaction. According to Institutional policies for analysis of publicly available data, this study was considered exempt from Institutional Review Board (IRB) review. The study adhered to the Strengthening Reporting of Observational Studies in Epidemiology (STROBE) guidelines to ensure transparency of the study [16].

### Data abstraction

Data on mortality, including age, sex, race, state, census, urbanization, and location of death, were taken from 1999 to 2023. Medical facilities (inpatient, outpatient, emergency room, death on arrival), the decedent’s home, hospice facility, nursing home, or unknown.

The age group was divided into two categories: 45–64 and 65 years. Hispanic or Latino, Non-Hispanic (NH) White, NH Black, NH American Indian or Alaska Native, and NH Asian or Pacific Islander were the categories for race and ethnicity. The National Center for Health Statistics (NCHS) Urban-Rural Classification Scheme was used to classify urbanization, dividing nations into metropolitan and non-metropolitan categories [17]. The four geographic regions are categorized as South, Midwest, Northeast, and West.

### Statistical analysis

The age-adjusted mortality rate (AAMR) and crude mortality rate (CMR) per 100,000 were computed for deaths involving both Alzheimer’s disease and Diabetes Mellitus. By dividing the number of fatalities by the entire population of that year, the crude mortality rate is calculated. 95% CIs were supplied for AAMRs, which were standardized to the 2000 U.S. standard population [18].

The Join point Regression Program was used to identify temporal trends. It fits a log-linear regression model that detects significant trend changes and calculates Average Annual Percentage Changes (AAPCs) and Annual Percentage Changes (APCs) with 95% confidence intervals [19]. A p-value of less than 0.05 indicates that a trend is statistically significant.

## Results

### Annual trends for Alzheimer’s disease mortality in patients with Diabetes mellitus

**Fig. 1:**
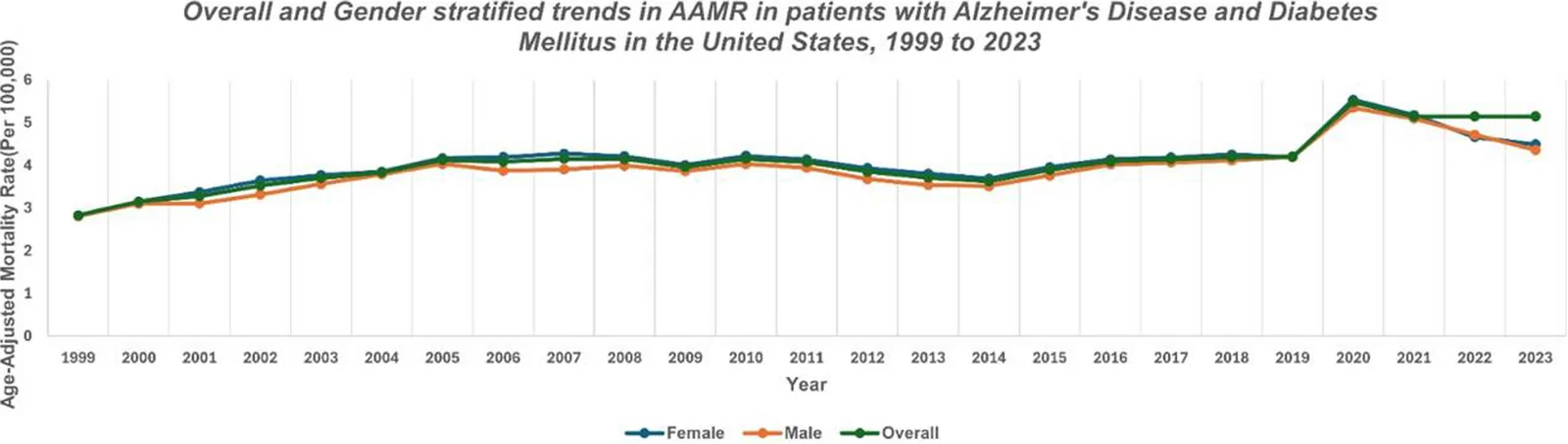
Gender-stratified trends in patients with AD and DM in the United States, 1999 to 2023

In individuals with both Alzheimer’s disease and Diabetes mellitus, the overall age-adjusted mortality rate (AAMR) rose from 2.82 (95% CI: 2.74 to 2.90) in 1999 to 4.42 (95% CI: 4.34 to 4.50) in 2023. A significant rise was observed in the trend between 1999 and 2023. From 1999 to 2005, there was a significant increase in mortality rate (APC: 6.00%; CI: 3.71 to 8.34 ; *p* < 0.05) than followed by an insignificant rise in trend from 2005 to 2017 (APC: -0.59%; CI: -1.31 to 0.12 ; *p* > 0.05) than, there was a significant increase in trend between 2017 and 2020 (APC: 10.87%; CI: 0.64 to 22.13 ; *p* < 0.05). Between 2020 and 2023, there was a significant decrease in the trend (APC: -5.18%; CI: -9.37 to -0.78; *p* < 0.05) (Tables 1 and 2, and Fig. 1**)**.

**Table 1:** Alzheimer’s disease with Diabetes mellitus-related deaths stratified by sex and race among middle-aged and older adults in the United States, 1999–2023

Year	Overall		Male	Female		American Indian or Alaska Native	Asian or Pacific Islander	Black or African American	White	Hispanic or Latino	Total Population
1999	4963		1762	3201		16	59	532	4163	185	180,408,769
2000	5588		1945	3643		10	53	574	4708	229	181,984,640
2001	5926		1976	3950		15	70	595	4954	285	184,305,128
2002	6448		2151	4297		15	75	702	5330	303	186,208,028
2003	6891		2374	4517		23	84	753	5662	355	188,090,429
2004	7207		2561	4646		18	96	770	5929	378	190,205,384
2005	7925		2799	5126		30	129	875	6441	439	192,551,384
2006	8007		2753	5254		26	136	864	6522	445	195,019,359
2007	8325		2879	5446		37	167	920	6697	500	197,403,777
2008	8481		3012	5469		29	188	897	6816	540	199,795,090
2009	8232		2982	5250		39	197	923	6499	558	202,107,016
2010	8789		3159	5630		41	239	928	6949	617	203,891,983
2011	8874		3213	5661		39	264	949	6912	695	206,592,936
2012	8564		3089	5475		38	240	936	6658	677	208,826,037
2013	8473		3071	5402		44	236	882	6540	759	211,085,314
2014	8449		3138	5311		46	258	904	6402	823	213,809,280
2015	9249		3461	5788		51	315	978	6926	954	216,553,817
2016	9973		3793	6180		54	328	1134	7470	977	218,641,417
2017	10,307		3957	6350		50	355	1127	7774	985	221,447,331
2018	10,712		4135	6577		51	391	1110	7981	1157	223,311,190
2019	10,937		4331	6606		43	398	1148	8125	1208	224,981,167
2020	14,510		5674	8836		80	579	1729	10,328	1779	226,635,013
2021	12,830		5128	7702		97	521	1451	10,703	1517	228,238,412
2022	12,580		5021	7559		65	564	1343	10,556	1562	229,508,599
2023	11,842		4753	7089		69	521	1281	9908	1342	231,529,762
Total	224,082		83,117	140,965		1026	6463	24,305	176,953	19,269	5,163,131,262

**Table 2:** Annual percent change (APC) of Alzheimer’s disease with Diabetes mellitus-related age-adjusted mortality rate among middle-aged and older adults in the United States, 1999–2023

Year Interval	APC		Lower 95% CI	Upper 95% CI		*P*-value	
Overall							
1999–2005	6.0037*	3.7161			8.3418		0.000052
2005–2017	-0.5982	-1.3160			0.1247		0.097493
2017–2020	10.8724*	0.6498			22.1332		0.038180
2020–2023	-5.1800*	-9.3780			-0.7876		0.024533
1999–2023	1.7988*	0.4248			3.1916		0.010128
Male							
1999–2005	6.1190*	2.5245			9.8396		0.002394
2005–2017	-1.0432	-2.5751			0.5128		0.171392
2017–2020	6.0866*	2.8549			9.4199		0.001090
2020–2023	-8.3174	-19.8098			4.8220		0.186049
1999–2023	1.8177*	0.2165			3.4444		0.025920
Female							
1999–2005	6.2442*	3.9055			8.6354		0.000043
2005–2017	-0.6621	-1.4030			0.0844		0.077805
2017–2020	10.5563	-0.1719			22.4373		0.053471
2020–2023	-5.1233*	-9.5546			-0.4750		0.033432
1999–2023	1.7950*	0.3537			3.2571		0.014476
Middle-aged group							
1999–2023	1.5858*	0.3499			2.8369		0.014024
Older Adults							
1999–2005	6.0117*	3.7477			8.3250		0.000046
2005–2017	-0.5918	-1.3028			0.1244		0.000046
2017–2020	10.8211*	0.6814			21.9819		0.037594
2020–2023	-5.1698*	-9.3296			-0.8193		0.023655
1999–2023	1.7995*	0.4373			3.1801		0.009463
American Indian or Alaska Native							
2005–2023	-0.7798	-2.3854			0.8521		0.325510
Asian or Pacific Islander							
1999–2010	8.3133*	4.9792			11.7532		0.000049
2010–2013	-7.9496	-31.2179			23.1903		0.556582
2023–2023	3.3340*	1.4670			5.2353		0.001446
1999-023	4.0720*	0.2876			7.9991		0.034691
Black or African American							
1999–2005	7.6803*	4.1644			11.3150		0.000293
2005–2017	-1.6976*	-2.7593			-0.6243		0.004472
2017–2020	11.4928	-3.9297			29.3913		0.139377
2020–2023	-8.3856*	-14.4013			-1.9471		0.015169
1999–2023	1.2669	-0.8151			3.3926		0.234922
White							
1999–2005	6.1227*	3.1769			9.1527		0.000474
2005–2015	-1.3953*	-2.7068			-0.0661		0.040998
2015–2021	6.0672*	3.0766			9.1445		0.000585
2021–2023	-6.0902	-16.4456			5.5486		0.268026
1999–2023	1.8663*	0.4747			3.2772		0.008420
Hispanic or Latino							
1999–2005	8.0153*	4.4010			11.7548		0.000253
2005–2017	0.7774	-0.0854			1.6477		0.073895
2017–2020	13.5590*	3.2070			24.9494		0.012772
2020–2023	-7.7308*	-11.6581			-3.6290		0.001402
1999–2023	2.9409*	1.4260			4.4784		0.000127
Census Region 1: Northeast							
1999–2005	2.8434*	0.6649			5.0690		0.013941
2005–2017	-2.1237*	-2.9203			-1.3206		0.000062
2017–2020	11.4409	-0.6391			24.9895		0.062381
2020–2023	-7.6432*	-12.6826			-2.3129		0.008836
1999–2023	-0.0122	-1.5829			1.5836		0.987959
Census Region 2: Midwest							
1999–2005	6.9822*	5.1948			8.7999		0.000001
2005–2015	-1.5648*	-2.3450			-0.7784		0.000806
2015–2020	6.7397*	4.0780			9.4696		0.000073
2020–2023	-5.5014*	-9.0363			-1.8292		0.006636
1999–2023	1.6959*	0.8726			2.5259		0.000050
Census Region 3: South							
1999–2007	4.8897*	2.5267			7.3072		0.000505
2007–2014	-3.3275*	2.5267			-0.2987		0.033784
2014–2021	6.0132*	3.2767			8.8222		0.000288
2021–2023	-6.9676	-19.4332			7.4268		0.299804
1999–2023	1.7202*	0.0192			3.4501		0.047447
Midwest region							
1999–2008	6.6800*	4.5477			8.8557		0.000008
2008–2018	0.5946	-0.1738			1.3689		0.119525
2018–2021	9.8938	-0.0471			20.8234		0.051002
2021–2023	-11.0068*	-19.0250			-2.1947		0.019071
1999–2023	2.4187*	0.9444			3.9146		0.001235
Metropolitan Areas							
1999–2005	-5.3766*	3.0272			7.7795		0.000201
2005–2008	-0.2428	-0.8862			0.4049		0.433957
2018–2020	15.9788*	5.7535			27.1927		0.003946
1999–2020	2.7960*	1.7110			3.8925		< 0.000001
Non-Metropolitan Areas							
1999–2007	5.8206*	3.6558			8.0307		0.000041
2007–2015	-2.4530*	-4.6272			-0.2292		0.033123
2015–2020	7.3566*	3.9103			10.9171		0.000364
1999–2020	2.9417*	1.6328			4.2675		0.000009

### Trends stratified by gender

Females consistently maintained higher Age-Adjusted Mortality Rates (AAMRs) across all years, with an overall AAMR of 4.01 compared to 3.86 among males.

In males, there was a significant increase in mortality trend from 1999 to 2005 (APC: 6.11%; CI: 2.52 to 9.83; *p* < 0.05), followed by an insignificant decrease in the trend from 2005 to 2015 (APC: -1.04%; CI: -2.57 to 0.51; *p* > 0.05). From 2015 to 2021, there was a gradual, significant increase in trend (APC: 6.08%; CI: 2.85 to 9.41; *p* > 0.05) followed by a steeper, insignificant decrease in trend from 2021 to 2025 (APC: -8.31%; CI: -19.80 to 4.82; *p* > 0.05).

In females, there was a significant increase in a trend from 1999 to 2005 (APC: 6.24%; CI: 3.90 to 8.63 ; *p* < 0.05), followed by a gradual, insignificant decrease in a trend from 2005 to 2017 (APC: -0.66%; CI: -1.40 to 0.08 ; *p* > 0.05). From 2017 to 2020, there was an insignificant increase in a trend (APC: 10.55%; CI: -0.17 to 22.43; *p* > 0.05) followed by a steeper, significant decline in a trend from 2020 to 2023 (APC: -5.12%; CI: -9.55 to -0.47; *p* < 0.05) **(**Table 2, Supplemental Tables 1, and Fig. 1**).**

### Trends stratified by age

Most age groups were showing an increase in mortality. In adults aged 45–64 years, the crude death rate rose from 0.09 in 1999–2020 to 0.15 in 2021–2023. In contrast, adults aged ≥ 65 years experienced a slower growth, however crude death rate remain elevated from 19.94 in 1999–2020 to 21.32 in 2021–2023. Overall, mortality showed an increasing trend in middle-aged and older adults.

Among the age group 45–64 years (middle age group), there was a significant increase in mortality trend from 1999 to 2023 (APC: 1.58%; CI: 0.34 to 2.83; *p* < 0.05).

**Fig. 2:**
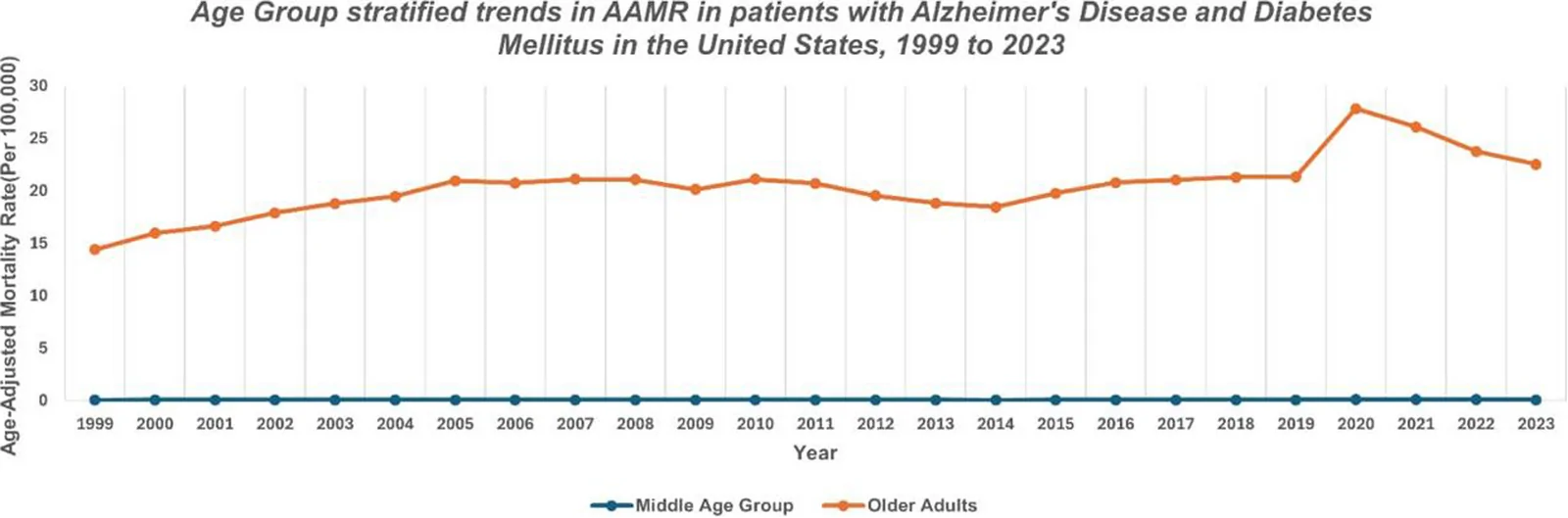
Age-stratified trends in patients with AD and DM in the United States, 1999 to 2023

Among the age group ≥ 65 years (older adults), there was a significantly steeper incline in a trend from 1999 to 2005 (APC: 6.01%; CI: 3.74 to 8.32 ; *p* < 0.05) followed by a gradual decrease in a trend from 2005 to 2017 (APC: -0.59%; CI: -1.30 to 0.12 ; *p* > 0.05). From 2017 to 2020, there was a significantly steeper incline in the trend (APC: 10.82%; CI: 0.68 to 21.98; *p* < 0.05), followed by an insignificant decrease in the trend from 2020 to 2023 (APC: -5.16%; CI: -9.32 to -0.81; *p* < 0.05) **(**Table 2, Supplemental Tables 2, and Fig. 2**).**

### Trends stratified by race

The Hispanic or Latino individuals accounted for 8.59% deaths, the vast majority of deaths occurred in non-Hispanic individuals (91.41%), with White individuals comprising the largest proportion (78.98%), followed by Black/African American (10.84%), Asian or Pacific Islander (2.88%), and American Indian or Alaska Native (0.45%).

Among Asian or Pacific Islander, there was a significant gradual increase in mortality trend from 1999 to 2010 (APC: 8.31%; CI: 4.97 to 11.75 ; *p* < 0.05) followed by an insignificant decrease in the trend from 2010 to 2013 (APC: -7.94%; CI: -31.21 to 23.19 ; *p* > 0.05). From 2013 to 2023, there was a significant gradual increase in a trend (APC: 3.33%; CI: 1.46 to 5.23; *p* < 0.05).

Among Black/African American, there was a significant increase in a mortality trend from 1999 to 2005 (APC: 7.68%; CI: 4.16 to 11.31 ; *p* < 0.05) followed by a significant decrease in a trend from 2005 to 2017 (APC: -1.69%; CI: -2.75 to -0.62 ; *p* < 0.05). From 2017 to 2020, there was an insignificant increase in trend (APC: 11.49%; CI: -3.92 to 29.39; *p* > 0.05) followed by a significant decrease in trend from 2020 to 2023 (APC: -8.38%; CI: -14.40 to -1.94; *p* < 0.05).

Among White individuals, there was a similar trend. From 1999 to 2005, there was a significant increase in the trend (APC: 6.12%; CI: 3.17 to 9.15; *p* < 0.05), followed by a significant gradual decrease in the trend from 2005 to 2015 (APC: -1.39%; CI: -2.70 to -0.06; *p* < 0.05). From 2015 to 2021, there was a significant increase in a trend (APC: 6.06%; CI: 3.07 to 9.14; *p* < 0.05) followed by an insignificant decrease in a trend from 2021 to 2023 (APC: -6.09%; CI: -16.44 to 5.54; *p* > 0.05).

**Fig. 3:**
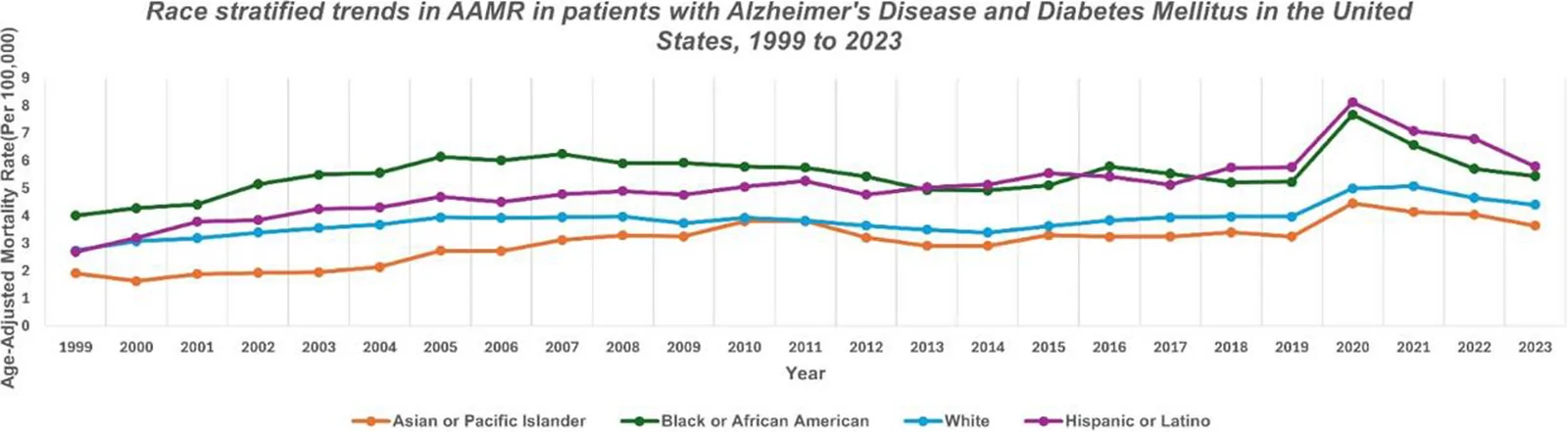
Race-stratified trends in patients with AD and DM in the United States, 1999 to 2023

Among Hispanic individuals, there was a progressive increase followed by a decrease in the trend. From 1999 to 2005, there was a significant increase in a trend (APC: 8.01%; CI: 4.40 to 11.75; *p* < 0.05) followed by an insignificant gradual increase in a trend from 2005 to 2017 (APC: 0.77%; CI: -0.08 to 1.64; *p* > 0.05). From 2017 to 2020, there was a significantly steeper incline in the trend (APC: 13.55%; CI: 3.20 to 24.94; *p* < 0.05), followed by a significant decrease in the mortality trend from 2020 to 2023 (APC: -7.73%; CI: -11.65 to -3.62; *p* < 0.05) **(**Table 2, Supplemental Tables 3, and Fig. 3**). **In American Indian or Alaska Native populations, the mortality data were unreliable.

### Trends stratified by geographic region

The highest AAMR was observed in the West (4.66; 95% CI: 4.46–4.86), followed by the Midwest (4.28; 95% CI: 4.09–4.46), then the South (4.20; 95% CI: 4.06–4.35), followed by the Northeast (2.66; 95% CI: 2.50–2.81).

In the West region, there was a significant increase in a trend from 1999 to 2006 (APC: 6.68%; CI: 4.54 to 8.85; *p* < 0.05) followed by an insignificant gradual increase in a trend from 2006 to 2018 (APC: 0.59%; CI: -0.17 to 1.36; *p* > 0.05). From 2018 to 2021, there was an insignificant increase in trend (APC: 9.89%; CI: -0.04 to 20.82; *p* > 0.05) followed by a steeper and significant decrease in the trend from 2021 to 2023 (APC: -11.01%; CI: -19.02 to -2.19; *p* < 0.05).

In the Midwest region, there is a significant gradual increase in trend from 1999 to 2005 (APC: 6.98%; CI: 5.19 to 8.79; *p* < 0.05), followed by a significant gradual decrease from 2005 to 2015 (APC: -1.56%; CI: -2.34 to -0.77; *p* < 0.05). From 2015 to 2020, there was a significant increase in a trend (APC: 6.73%; CI: 4.07 to 9.46; *p* < 0.05) followed by a significant decrease in trend from 2020 to 2023 (APC: -5.50%; CI: -9.03 to -1.82; *p* < 0.05).

In the South region, a similar trend is observed. From 1999 to 2007, there was a significant increase in trend (APC: 4.88%; CI: 2.52 to 7.30; *p* < 0.05) followed by a significant decrease in trend from 2007 to 2014 (APC: -3.32%; CI: -6.26 to -0.2; *p* < 0.05). From 2014 to 2021, there was a significant increase in the trend (APC: 6.01%; CI: 3.27 to 8.82; *p* < 0.05) followed by a gradual, insignificant decline in the trend (APC: -6.96%; CI: -19.43 to 7.42; *p* > 0.05).

**Fig. 4:**
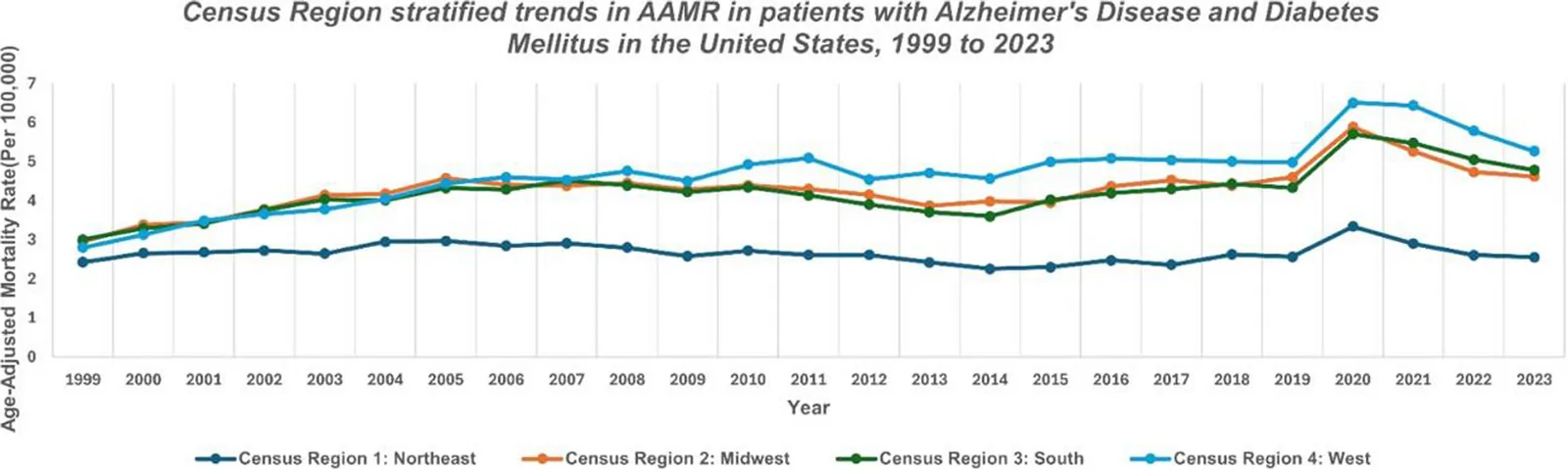
Region-stratified trends in patients with AD and DM in the United States, 1999 to 2023

In the Northeast region, a similar trend is observed. From 1999 to 2005, there was a significant increase in the mortality trend (APC: 2.84; CI: 0.66 to 5.06; *p* < 0.05) followed by a significant and gradual decrease in the trend from 2005 to 2017 (APC: -2.12%; CI: -2.92 to -1.32; *p* < 0.05). From 2017 to 2020, there was an insignificant increase in trend (APC: 11.44%; CI: -0.63 to 24.98; *p* > 0.05) followed by a significant, gradual decrease in trend from 2020 to 2023 (APC: -7.64%; CI: -12.68 to -2.31; *p* < 0.05) **(**Table 2, Supplemental Tables 4, and Fig. 4**).**

### Trends stratified by urbanization

From 1999 to 2020, we observed that non-metropolitan areas consistently had higher age-adjusted mortality rates (AAMRs) compared to metropolitan areas throughout the study period.

For Metropolitan areas, there is an increase followed by a decrease, followed by an increase in the trend. From 1999 to 2005 there was a significant increase in a trend (APC: 5.37%; CI: 3.02 to 7.77; *p* < 0.05) followed by an insignificant decrease in a trend from 2005 to 2018 (APC: -0.24%; CI: -0.88 to 0.40 ; *p* > 0.05) than there is a significant increase in a trend from 2018 to 2020 (APC: 15.97%; CI: 5.75 to 27.19 ; *p* < 0.05).

**Fig. 5:**
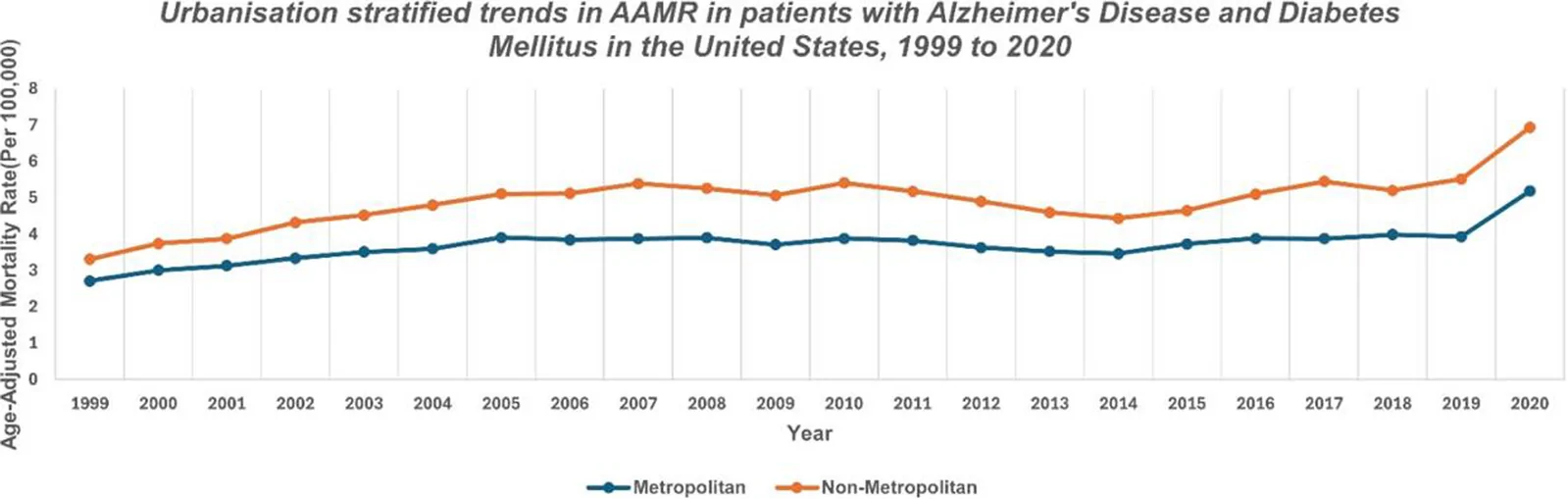
Urbanization-stratified trends in patients with AD and DM in the United States, 1999 to 2023

For non-metropolitan areas, a similar trend is observed. From 1999 to 2007 there is a significant and gradual increase in a trend (APC: 5.82%; CI: 3.65 to 8.03 ; *p* < 0.05) followed by a significant decrease in a trend from 2007 to 2015 (APC: -2.45%; CI: -4.62 to -0.22 ; *p* < 0.05) than there was a significant increase in mortality trend from 2015 to 2020 (APC: 7.35%; CI: 3.91 to 10.91 ; *p* < 0.05) **(**Table 2, Supplemental Tables 5, and Fig. 5**). **

### States data

From 1999 to 2020, AAMRs ranged from as low as 1.68 (95% CI:1.53–1.82) in Nevada to as high as 7.03 (95% CI: 6.77–7.28) in Mississippi. States falling within the top 90th percentile included: Mississippi (7.03), North Dakota (6.40), Washington (6.20), Vermont (6.12), and Tennessee (5.95) which had overall higher AAMR than the states in lower 10th percentile Nevada (1.68), Florida (1.92), New York (1.94), Massachusetts (2.27), Connecticut (2.50).

From 2021 to 2023, AAMRs ranged from as low as 1.54 (95% CI:1.29–1.79) in Connecticut to as high as 12.72 (95% CI: 11.85–13.58) in Mississippi. States falling within the top 90th percentile included: Mississippi (12.72), Oklahoma (9.80), South Carolina (8.46), South Dakota (8.36), and Oregon (8.35) which had overall higher AAMR than the states in lower 10th percentile Connecticut (1.54), New Jersey (2.04), Florida (2.08), New York (2.29), and District of Columbia (2.42) **(**Supplemental Fig. 1a and 1b**).**

## Discussion

In this national, population-level analysis of CDC WONDER data (1999–2023), we document 224,082 deaths among U.S. adults aged ≥ 45 years with both diabetes mellitus and Alzheimer’s disease (AD), of whom women accounted for two-thirds of deaths (62.9%). The age-adjusted mortality rate (AAMR) rose from 2.82 to 4.42 per 100,000 over the study period, but the temporal trend was non-linear with phases of accelerating (1999–2005; 2017–2020) and an interim decline in 2020–2023. These overall characteristics are consistent with recent estimates having reported increasing AD-related mortality in individuals with diabetes and increasing population burdens of AD that also reflect period-specific changes and disruptions (e.g., pandemic effects) [4, 12, 20].

Women made up a disproportionately high proportion of deaths. This is consistent with known epidemiologic patterns in which women have higher AD prevalence and lifetime risk because of greater survival, sex-specific biology (e.g., menopausal-related changes, hormonal and immunologic differences), and slopes of cognitive decline differences. These mechanisms—and concomitant vascular comorbidity and competing cause of death sex differences in men (men’s preclinical cardiovascular mortality)—presumably are responsible for the observed excess female mortality among the AD + diabetes cohort [21].

Statistically significant mortality increases in 45–64-year-olds is concerning and consistent with findings that midlife diabetes hugely increases subsequent risk of dementia and dementia mortality; earlier age and longer duration of diabetes multiply neurodegenerative process through hyperglycemia, insulin resistance, ad vascular injury [12, 22]. Our findings support this interpretation, and midlife metabolic risk therefore is a critical time for prevention.

Recent data indicate a statistically significant rise in mortality among adults aged 45–64. This is in concert with epidemiologic evidence that midlife diabetes markedly increases later dementia risk and mortality. For instance, one large twin-cohort study estimated that diabetes doubles dementia risk, with the strongest effect when diabetes begins before age 65 [22]. Similarly, population data indicate that younger age at diabetes onset and longer diabetes duration accelerate neurodegeneration. Thus, one Australian study estimated that diabetic subjects developed dementia ~ 2.2 years earlier and died ~ 2.6 years sooner than nondiabetics; subjects with ≥ 15-year diabetes histories and early-onset dementia had nearly twice the mortality rate of nondiabetics (HR ≈ 1.9) [23]. Together, these findings support the idea that midlife metabolic health represent a critical window for prevention include screening for diabetes, glycemic control, lifestyle modifications and reducing the risk of vascular injury lowering the risk for dementia.

Alzheimer’s-related deaths remain highest among non‐Hispanic whites, but temporal patterns differ markedly by race/ethnicity. For example, whites have the largest burden of AD mortality, but Black and Hispanic populations have different growth rates. Such heterogeneity will depend on many factors to include genetic risks (e.g., APOE ε4 prevalence), comorbidities, and social determinants. Notably, vascular risk factors vary by group: hypertension and diabetes are more prevalent in some communities. For instance, hypertension has been cited as a major contributor to higher AD risk in Black compared with White populations. Access to care and diagnostic practices also vary neurology specialists commonly are lacking within rural and socioeconomically disadvantaged areas, which contributes to higher age-adjusted AD mortality in non‐metropolitan regions (where vascular disease also is more common and resources scarcer). In sum, differences in risk‐factor profiles, healthcare access, and socioeconomic context help explain the observed racial/ethnic and urban–rural disparities in dementia mortality. Regional variation and larger non-metropolitan AAMRs likely arise from unequal access to specialist neurology care, higher burden of vascular risk, and socioeconomic disparities [4, 24].

The fall in AAMR after 2020 can partly be explained by pandemic distortions: (1) competing mortality causes (COVID-19 pandemic) generating substitute primary cause assignment; (2) disruption in chronic-disease care and diagnostic determination; and (3) changes in healthcare use and death certification during the pandemic era. The COVID-19 pandemic distorted mortality patterns after 2020. Much of the apparent fall in AD-related mortality is owing to competing mortality and reporting changes. In the first pandemic year, the COVID-19 pandemic directly or indirectly accounted for many deaths that otherwise might have been attributed to chronic causes. For example, one state-level analysis estimated that dementia accounted for 16.4% of the additional deaths associated with the COVID-19 pandemic in 2020 [25]. At the same time, healthcare disruptions - lockdowns, delayed care, testing shortages - along with changed death certification practices led to undercounting or misclassification of dementia deaths. These factors mean the post-2020 decline in AD mortality rates needs to be interpreted with caution; it likely reflects pandemic-related reporting and care, rather than a true improvement in dementia outcomes [25–27]. Caution is therefore required to interpret such a fall as an actual reduction in AD-related mortality.

Diabetes accelerates AD development through synergistic processes—vascular injury, inflammation, insulin resistance, and disordered cerebral glucose metabolism—thereby augmenting mortality hazard over additive impacts [7, 15, 28, 29]. In addition, increased survival from cardiovascular illness over recent decades may paradoxically raise the number exposed to AD mortality (a competing-risk/survivor effect) [28, 30, 31].

### Strengths and limitations

Strengths include large national sample size and extended study duration to permit join point analyses and subgroup stratification. Weaknesses include deaths-certificate coding dependency (potential misclassification), lack of individual-level data on diabetes duration, glycemic control, AD severity, and potential pandemic artifacts on cause assignment. These limitations limit causal inference and emphasize the need for linked clinical registry and EHR studies [24, 29].

### Implications

The results highlight the need for midlife prevention and control of diabetes as part of risk-reduction strategies for dementia, sex-specific studies and care pathways, and targeted interventions to bridge geographic and racial disparities—especially in non-metropolitan and underserved populations. Prospective cohort studies that relate diabetes courses, AD biomarkers, and cause-of-death analyses will be critical to untangle causal pathways and inform targeted interventions [12, 22, 23].

## Conclusions

Mortality associated with both Alzheimer’s disease and diabetes mellitus has increased over the time with disparities in sex, gender, race, and geographical distributions. The mortality rise post 2020 may reflect changes associated with the COVID-19 pandemic. These findings underscore the need for preventing strategies targeting diabetes and vascular risk factors.

However, Alzheimer cannot be fully prevented but effective diabetes prevention and management through glycemic control, smoking cessation, cardiovascular risk reduction, and other lifestyle modifications can reduce mortality related to dementia.

## Supplementary Information

Supplementary Material 1.

## Data Availability

The datasets generated and/or analyzed during the current study are available from the corresponding author on reasonable request.

## Abbreviations

AD: Alzheimer’s Disease
DM: Diabetes Mellitus
CDC: Centers for Disease Control and Prevention
WONDER: Wide–Ranging Online Data for Epidemiologic Research
MCOD: Multiple Cause of Death
ICD: 10–International Classification of Diseases, 10th Revision
AAMR: Age–Adjusted Mortality Rate
CMR: Crude Mortality Rate
APC: Annual Percentage Change
AAPC: Average Annual Percentage Change
CI: Confidence Interval
STROBE: Strengthening the Reporting of Observational Studies in Epidemiology
