# Supplementary material for: Trends in both Alzheimer’s disease and Diabetes mellitus related mortality among middle-aged and older adults in the United States, 1999 to 2023: a CDC WONDER database analysis
Source: BMC Neurol. 2026 Jun 12;26:638. doi: 10.1186/s12883-026-05058-2 (PMC13651194; doi:10.1186/s12883-026-05058-2)
Supplement: Supplementary file 1 [file 12883_2026_5058_MOESM1_ESM.docx]

**SUPPLEMENTAL FIGURES:**

**Supplementary Figure 01: Gender-stratified trends in patients with AD and DM in the United States, 1999 to 2023**

**Supplementary Figure 02: Age-stratified trends in patients with AD and DM in the United States, 1999 to 2023**

**Supplementary Figure 03: Race-stratified trends in patients with AD and DM in the United States, 1999 to 2023**

**Supplementary Figure 04: Region-stratified trends in patients with AD and DM in the United States, 1999 to 2023**

**Supplementary Figure 05: Urbanization-stratified trends in patients with AD and DM in the United States, 1999 to 2023**

Gender stratified trends in AAMR in patients with Alzheimer's Disease in the United States, 1999 to 2023

Age-Adjusted Mortality Rate(Per 100,000)

| Year | Female | Male | Overall |
| --- | --- | --- | --- |
| Year | 46.238 | 41.7267 | 45.0114 |
| 1999 | 49.4283 | 42.7389 | 47.3862 |
| 2000 | 51.4453 | 43.8848 | 49.0977 |
| 2001 | 55.2776 | 46.4361 | 52.4735 |
| 2002 | 56.4417 | 47.1287 | 53.4568 |
| 2003 | 56.8351 | 47.0789 | 53.6241 |
| 2004 | 59.3566 | 48.2721 | 55.7299 |
| 2005 | 57.7035 | 46.9521 | 54.0947 |
| 2006 | 57.2468 | 46.0523 | 53.433 |
| 2007 | 57.7419 | 46.2308 | 53.7748 |
| 2008 | 53.6591 | 43.5952 | 50.1354 |
| 2009 | 54.6956 | 44.631 | 51.1814 |
| 2010 | 53.3602 | 42.4272 | 49.4951 |
| 2011 | 50.7712 | 40.6035 | 47.1151 |
| 2012 | 50.0848 | 39.2842 | 46.1839 |
| 2013 | 53.3525 | 40.7256 | 48.7015 |
| 2014 | 60.8281 | 46.1588 | 55.4133 |
| 2015 | 62.8511 | 47.5309 | 57.1644 |
| 2016 | 64.1066 | 48.166 | 58.1267 |
| 2017 | 63.0365 | 47.4387 | 57.1316 |
| 2018 | 61.7762 | 45.9912 | 55.7451 |
| 2019 | 72.6796 | 53.1356 | 65.1395 |
| 2020 | 67.8533 | 49.2269 | 60.5509 |
| 2021 | 61.7469 | 46.3868 | 55.8003 |
| 2022 | 59.2001 | 42.7628 | 52.7204 |

**Supplementary Figure 06: Gender-stratified trends in patients with AD in the United States, 1999 to 2023**

Gender stratified trends in AAMR in patients with Diabetes Mellitus in the United States, 1999 to 2023

Age-Adjusted Mortality Rate(Per 100,000)

| Year | Female | Male | Overall |
| --- | --- | --- | --- |
| 1999 | 104.881 | 137.716 | 118.467 |
| 2000 | 104.866 | 138.863 | 118.987 |
| 2001 | 105.388 | 139.812 | 119.809 |
| 2002 | 105.484 | 142.886 | 121.116 |
| 2003 | 104.609 | 143.359 | 120.939 |
| 2004 | 101.647 | 140.404 | 117.989 |
| 2005 | 102.86 | 143.09 | 119.898 |
| 2006 | 99.2345 | 139.278 | 116.318 |
| 2007 | 97.0337 | 137.178 | 114.242 |
| 2008 | 95.6914 | 136.026 | 113.037 |
| 2009 | 91.504 | 132.692 | 109.331 |
| 2010 | 90.9697 | 133.034 | 109.127 |
| 2011 | 90.382 | 132.227 | 108.577 |
| 2012 | 87.9512 | 130.973 | 106.74 |
| 2013 | 87.4381 | 131.415 | 106.748 |
| 2014 | 83.9752 | 128.381 | 103.518 |
| 2015 | 84.3875 | 129.655 | 104.392 |
| 2016 | 83.8 | 130.967 | 104.646 |
| 2017 | 85.1464 | 134.17 | 106.878 |
| 2018 | 84.0639 | 134.835 | 106.604 |
| 2019 | 83.8953 | 135.716 | 106.998 |
| 2020 | 112.671 | 183.679 | 144.479 |
| 2021 | 119.261 | 190.736 | 151.395 |
| 2022 | 105.382 | 171.301 | 134.646 |
| 2023 | 95.6133 | 155.734 | 122.614 |

**Supplementary Figure 07: Gender-stratified trends in patients with DM in the United States, 1999 to 2023**

**SUPPLEMENTAL TABLES:**

| **Supplementary Table 01:** **Alzheimer's Disease with Diabetes Mellitus-related Deaths Stratified by Sex and Race among middle-aged and older adults in the United States, 1999-2023** | | | | | | | | | |
| --- | --- | --- | --- | --- | --- | --- | --- | --- | --- |
| **Year** | **Overall** | **Male** | **Female** | **American Indian or Alaska Native** | **Asian or Pacific Islander** | **Black or African American** | **White** | **Hispanic or Latino** | **Total Population** |
| **1999** | 4963 | 1762 | 3201 | 16 | 59 | 532 | 4163 | 185 | 180408769 |
| **2000** | 5588 | 1945 | 3643 | 10 | 53 | 574 | 4708 | 229 | 181984640 |
| **2001** | 5926 | 1976 | 3950 | 15 | 70 | 595 | 4954 | 285 | 184305128 |
| **2002** | 6448 | 2151 | 4297 | 15 | 75 | 702 | 5330 | 303 | 186208028 |
| **2003** | 6891 | 2374 | 4517 | 23 | 84 | 753 | 5662 | 355 | 188090429 |
| **2004** | 7207 | 2561 | 4646 | 18 | 96 | 770 | 5929 | 378 | 190205384 |
| **2005** | 7925 | 2799 | 5126 | 30 | 129 | 875 | 6441 | 439 | 192551384 |
| **2006** | 8007 | 2753 | 5254 | 26 | 136 | 864 | 6522 | 445 | 195019359 |
| **2007** | 8325 | 2879 | 5446 | 37 | 167 | 920 | 6697 | 500 | 197403777 |
| **2008** | 8481 | 3012 | 5469 | 29 | 188 | 897 | 6816 | 540 | 199795090 |
| **2009** | 8232 | 2982 | 5250 | 39 | 197 | 923 | 6499 | 558 | 202107016 |
| **2010** | 8789 | 3159 | 5630 | 41 | 239 | 928 | 6949 | 617 | 203891983 |
| **2011** | 8874 | 3213 | 5661 | 39 | 264 | 949 | 6912 | 695 | 206592936 |
| **2012** | 8564 | 3089 | 5475 | 38 | 240 | 936 | 6658 | 677 | 208826037 |
| **2013** | 8473 | 3071 | 5402 | 44 | 236 | 882 | 6540 | 759 | 211085314 |
| **2014** | 8449 | 3138 | 5311 | 46 | 258 | 904 | 6402 | 823 | 213809280 |
| **2015** | 9249 | 3461 | 5788 | 51 | 315 | 978 | 6926 | 954 | 216553817 |
| **2016** | 9973 | 3793 | 6180 | 54 | 328 | 1134 | 7470 | 977 | 218641417 |
| **2017** | 10307 | 3957 | 6350 | 50 | 355 | 1127 | 7774 | 985 | 221447331 |
| **2018** | 10712 | 4135 | 6577 | 51 | 391 | 1110 | 7981 | 1157 | 223311190 |
| **2019** | 10937 | 4331 | 6606 | 43 | 398 | 1148 | 8125 | 1208 | 224981167 |
| **2020** | 14510 | 5674 | 8836 | 80 | 579 | 1729 | 10328 | 1779 | 226635013 |
| **2021** | 12830 | 5128 | 7702 | 97 | 521 | 1451 | 10703 | 1517 | 228238412 |
| **2022** | 12580 | 5021 | 7559 | 65 | 564 | 1343 | 10556 | 1562 | 229508599 |
| **2023** | 11842 | 4753 | 7089 | 69 | 521 | 1281 | 9908 | 1342 | 231529762 |
| **Total** | 224,082 | 83,117 | 140,965 | 1026 | 6463 | 24,305 | 176,953 | 19,269 | 5,163,131,262 |

| **Supplemental Table 02: Annual percent change (APC) of Alzheimer's Disease with Diabetes Mellitus-related Age-Adjusted Mortality Rate among middle-aged and older adults in the United States, 1999-2023** | | | | | | | |
| --- | --- | --- | --- | --- | --- | --- | --- |
| **Year Interval** | **APC** | | **Lower 95%  CI** | **Upper 95%  CI** | | **P-value** | |
| **Overall** | | | | | | | |
| **1999-2005** | 6.0037* | 3.7161 | | | 8.3418 | | 0.000052 |
| **2005-2017** | -0.5982 | -1.3160 | | | 0.1247 | | 0.097493 |
| **2017-2020** | 10.8724* | 0.6498 | | | 22.1332 | | 0.038180 |
| **2020-2023** | -5.1800* | -9.3780 | | | -0.7876 | | 0.024533 |
| **1999-2023** | 1.7988* | 0.4248 | | | 3.1916 | | 0.010128 |
| **Male** | | | | | | | |
| **1999-2005** | 6.1190* | 2.5245 | | | 9.8396 | | 0.002394 |
| **2005-2017** | -1.0432 | -2.5751 | | | 0.5128 | | 0.171392 |
| **2017-2020** | 6.0866* | 2.8549 | | | 9.4199 | | 0.001090 |
| **2020-2023** | -8.3174 | -19.8098 | | | 4.8220 | | 0.186049 |
| **1999-2023** | 1.8177* | 0.2165 | | | 3.4444 | | 0.025920 |
| **Female** | | | | | | | |
| **1999-2005** | 6.2442* | 3.9055 | | | 8.6354 | | 0.000043 |
| **2005-2017** | -0.6621 | -1.4030 | | | 0.0844 | | 0.077805 |
| **2017-2020** | 10.5563 | -0.1719 | | | 22.4373 | | 0.053471 |
| **2020-2023** | -5.1233* | -9.5546 | | | -0.4750 | | 0.033432 |
| **1999-2023** | 1.7950* | 0.3537 | | | 3.2571 | | 0.014476 |
| **Middle-aged group** | | | | | | | |
| **1999-2023** | 1.5858* | 0.3499 | | | 2.8369 | | 0.014024 |
| **Older Adults** | | | | | | | |
| **1999-2005** | 6.0117* | 3.7477 | | | 8.3250 | | 0.000046 |
| **2005-2017** | -0.5918 | -1.3028 | | | 0.1244 | | 0.000046 |
| **2017-2020** | 10.8211* | 0.6814 | | | 21.9819 | | 0.037594 |
| **2020-2023** | -5.1698* | -9.3296 | | | -0.8193 | | 0.023655 |
| **1999-2023** | 1.7995* | 0.4373 | | | 3.1801 | | 0.009463 |
| **American Indian or Alaska Native** | | | | | | | |
| **2005-2023** | -0.7798 | -2.3854 | | | 0.8521 | | 0.325510 |
| **Asian or Pacific Islander** | | | | | | | |
| **1999-2010** | 8.3133* | 4.9792 | | | 11.7532 | | 0.000049 |
| **2010-2013** | -7.9496 | -31.2179 | | | 23.1903 | | 0.556582 |
| **2023-2023** | 3.3340* | 1.4670 | | | 5.2353 | | 0.001446 |
| **1999-023** | 4.0720* | 0.2876 | | | 7.9991 | | 0.034691 |
| **Black or African American,** | | | | | | | |
| **1999-2005** | 7.6803* | 4.1644 | | | 11.3150 | | 0.000293 |
| **2005-2017** | -1.6976* | -2.7593 | | | -0.6243 | | 0.004472 |
| **2017-2020** | 11.4928 | -3.9297 | | | 29.3913 | | 0.139377 |
| **2020-2023** | -8.3856* | -14.4013 | | | -1.9471 | | 0.015169 |
| **1999-2023** | 1.2669 | -0.8151 | | | 3.3926 | | 0.234922 |
| **White** | | | | | | | |
| **1999-2005** | 6.1227* | 3.1769 | | | 9.1527 | | 0.000474 |
| **2005-2015** | -1.3953* | -2.7068 | | | -0.0661 | | 0.040998 |
| **2015-2021** | 6.0672* | 3.0766 | | | 9.1445 | | 0.000585 |
| **2021-2023** | -6.0902 | -16.4456 | | | 5.5486 | | 0.268026 |
| **1999-2023** | 1.8663* | 0.4747 | | | 3.2772 | | 0.008420 |
| **Hispanic or Latino** | | | | | | | |
| **1999-2005** | 8.0153* | 4.4010 | | | 11.7548 | | 0.000253 |
| **2005-2017** | 0.7774 | -0.0854 | | | 1.6477 | | 0.073895 |
| **2017-2020** | 13.5590* | 3.2070 | | | 24.9494 | | 0.012772 |
| **2020-2023** | -7.7308* | -11.6581 | | | -3.6290 | | 0.001402 |
| **1999-2023** | 2.9409* | 1.4260 | | | 4.4784 | | 0.000127 |
| **Census Region 1: Northeast** | | | | | | | |
| **1999-2005** | 2.8434* | 0.6649 | | | 5.0690 | | 0.013941 |
| **2005-2017** | -2.1237* | -2.9203 | | | -1.3206 | | 0.000062 |
| **2017-2020** | 11.4409 | -0.6391 | | | 24.9895 | | 0.062381 |
| **2020-2023** | -7.6432* | -12.6826 | | | -2.3129 | | 0.008836 |
| **1999-2023** | -0.0122 | -1.5829 | | | 1.5836 | | 0.987959 |
| **Census Region 2: Midwest** | | | | | | | |
| **1999-2005** | 6.9822* | 5.1948 | | | 8.7999 | | 0.000001 |
| **2005-2015** | -1.5648* | -2.3450 | | | -0.7784 | | 0.000806 |
| **2015-2020** | 6.7397* | 4.0780 | | | 9.4696 | | 0.000073 |
| **2020-2023** | -5.5014* | -9.0363 | | | -1.8292 | | 0.006636 |
| **1999-2023** | 1.6959* | 0.8726 | | | 2.5259 | | 0.000050 |
| **Census Region 3: South** | | | | | | | |
| **1999-2007** | 4.8897* | 2.5267 | | | 7.3072 | | 0.000505 |
| **2007-2014** | -3.3275* | 2.5267 | | | -0.2987 | | 0.033784 |
| **2014-2021** | 6.0132* | 3.2767 | | | 8.8222 | | 0.000288 |
| **2021-2023** | -6.9676 | -19.4332 | | | 7.4268 | | 0.299804 |
| **1999-2023** | 1.7202* | 0.0192 | | | 3.4501 | | 0.047447 |
| **Midwest region** | | | | | | | |
| **1999-2008** | 6.6800* | 4.5477 | | | 8.8557 | | 0.000008 |
| **2008-2018** | 0.5946 | -0.1738 | | | 1.3689 | | 0.119525 |
| **2018-2021** | 9.8938 | -0.0471 | | | 20.8234 | | 0.051002 |
| **2021-2023** | -11.0068* | -19.0250 | | | -2.1947 | | 0.019071 |
| **1999-2023** | 2.4187* | 0.9444 | | | 3.9146 | | 0.001235 |
| **Metropolitan Areas** | | | | | | | |
| **1999-2005** | -5.3766* | 3.0272 | | | 7.7795 | | 0.000201 |
| **2005-2008** | -0.2428 | -0.8862 | | | 0.4049 | | 0.433957 |
| **2018-2020** | 15.9788* | 5.7535 | | | 27.1927 | | 0.003946 |
| **1999-2020** | 2.7960* | 1.7110 | | | 3.8925 | | < 0.000001 |
| **Non-Metropolitan Areas** | | | | | | | |
| **1999-2007** | 5.8206* | 3.6558 | | | 8.0307 | | 0.000041 |
| **2007-2015** | -2.4530* | -4.6272 | | | -0.2292 | | 0.033123 |
| **2015-2020** | 7.3566* | 3.9103 | | | 10.9171 | | 0.000364 |
| **1999-2020** | 2.9417* | 1.6328 | | | 4.2675 | | 0.000009 |

| **Supplemental Table 03: Overall and Sex-stratified Alzheimer's Disease with Diabetes Mellitus-related Age-Adjusted Mortality Rate among middle-aged and older adults in the United States, 1999-2023** | | | | | | | | | |
| --- | --- | --- | --- | --- | --- | --- | --- | --- | --- |
| **Year** | **Male** | | | **Female** | | | **Overall** | | |
|  | **AAMR** | **Lower 95%  CI** | **Upper 95%  CI** | **AAMR** | **Lower 95%  CI** | **Upper 95%  CI** | **AAMR** | **Lower 95%  CI** | **Upper 95%  CI** |
| **1999** | 2.812 | 2.6783 | 2.9457 | 2.8039 | 2.7063 | 2.9016 | 2.8272 | 2.7485 | 2.9059 |
| **2000** | 3.0972 | 2.9574 | 3.237 | 3.1484 | 3.0458 | 3.251 | 3.1449 | 3.0625 | 3.2274 |
| **2001** | 3.1044 | 2.9654 | 3.2434 | 3.3678 | 3.2623 | 3.4733 | 3.275 | 3.1916 | 3.3584 |
| **2002** | 3.3144 | 3.172 | 3.4567 | 3.6419 | 3.5325 | 3.7513 | 3.5234 | 3.4373 | 3.6094 |
| **2003** | 3.5619 | 3.4167 | 3.7071 | 3.7646 | 3.6543 | 3.8748 | 3.6991 | 3.6117 | 3.7865 |
| **2004** | 3.7857 | 3.6374 | 3.9341 | 3.8498 | 3.7385 | 3.9611 | 3.8329 | 3.7444 | 3.9214 |
| **2005** | 4.0292 | 3.8785 | 4.1798 | 4.1651 | 4.0504 | 4.2798 | 4.1207 | 4.0299 | 4.2115 |
| **2006** | 3.8697 | 3.7241 | 4.0152 | 4.1894 | 4.0752 | 4.3036 | 4.0819 | 3.9924 | 4.1714 |
| **2007** | 3.9008 | 3.7572 | 4.0443 | 4.273 | 4.1585 | 4.3876 | 4.1505 | 4.0612 | 4.2399 |
| **2008** | 3.9897 | 3.8464 | 4.133 | 4.2065 | 4.0937 | 4.3192 | 4.1433 | 4.0549 | 4.2318 |
| **2009** | 3.8606 | 3.7214 | 3.9998 | 3.9999 | 3.8903 | 4.1095 | 3.9588 | 3.8729 | 4.0446 |
| **2010** | 4.03 | 3.889 | 4.1709 | 4.2175 | 4.1057 | 4.3293 | 4.1524 | 4.0652 | 4.2396 |
| **2011** | 3.9403 | 3.8036 | 4.077 | 4.1323 | 4.0227 | 4.2418 | 4.0719 | 3.9866 | 4.1571 |
| **2012** | 3.6766 | 3.5465 | 3.8068 | 3.9283 | 3.8223 | 4.0343 | 3.8455 | 3.7634 | 3.9276 |
| **2013** | 3.536 | 3.4104 | 3.6617 | 3.7977 | 3.6944 | 3.9009 | 3.707 | 3.6274 | 3.7867 |
| **2014** | 3.5118 | 3.3883 | 3.6353 | 3.6833 | 3.5823 | 3.7844 | 3.6203 | 3.5424 | 3.6983 |
| **2015** | 3.7593 | 3.6334 | 3.8852 | 3.9534 | 3.8496 | 4.0573 | 3.8913 | 3.8113 | 3.9713 |
| **2016** | 4.0147 | 3.8861 | 4.1432 | 4.1361 | 4.0311 | 4.2411 | 4.0874 | 4.0064 | 4.1683 |
| **2017** | 4.0658 | 3.9384 | 4.1932 | 4.1776 | 4.0731 | 4.282 | 4.1398 | 4.0593 | 4.2203 |
| **2018** | 4.1127 | 3.9866 | 4.2387 | 4.2521 | 4.148 | 4.3563 | 4.1884 | 4.1086 | 4.2682 |
| **2019** | 4.1927 | 4.0672 | 4.3183 | 4.1889 | 4.0867 | 4.2911 | 4.1963 | 4.1172 | 4.2754 |
| **2020** | 5.3417 | 5.2018 | 5.4815 | 5.5314 | 5.415 | 5.6479 | 5.4785 | 5.389 | 5.5681 |
| **2021** | 5.0925 | 4.9516 | 5.2335 | 5.1743 | 5.058 | 5.2905 | 3.9604 | 3.9424 | 3.9785 |
| **2022** | 4.7135 | 4.5819 | 4.8452 | 4.6591 | 4.5534 | 4.7647 | 2.8272 | 2.7485 | 2.9059 |
| **2023** | 4.3565 | 4.2312 | 4.4818 | 4.4871 | 4.3824 | 4.5918 | 3.1449 | 3.0625 | 3.2274 |

| **Supplementary Table 04: Alzheimer's Disease with Diabetes Mellitus-related Deaths Stratified by Age among middle-aged and older adults in the United States, 1999-2023** | | | | | | |
| --- | --- | --- | --- | --- | --- | --- |
| **Year** | **Middle-aged group** | | | **Older adults** | | |
|  | **Crude Rate** | **Crude Rate Lower 95%  CI** | **Crude Rate Upper 95%  CI** | **Crude Rate** | **Crude Rate Lower 95%  CI** | **Crude Rate Upper 95%  CI** |
| **1999** | 0.058 | 0.0404 | 0.0806 | 14.1618 | 13.7664 | 14.5572 |
| **2000** | 0.071 | 0.0516 | 0.0953 | 15.8437 | 15.4267 | 16.2608 |
| **2001** | 0.0837 | 0.0629 | 0.1093 | 16.6391 | 16.2135 | 17.0647 |
| **2002** | 0.0855 | 0.0647 | 0.1107 | 17.9916 | 17.5505 | 18.4327 |
| **2003** | 0.0915 | 0.0703 | 0.1171 | 19.0388 | 18.5872 | 19.4904 |
| **2004** | 0.0677 | 0.0499 | 0.0897 | 19.7744 | 19.3164 | 20.2325 |
| **2005** | 0.0848 | 0.065 | 0.1087 | 21.449 | 20.9748 | 21.9231 |
| **2006** | 0.0904 | 0.0702 | 0.1146 | 21.3593 | 20.8894 | 21.8292 |
| **2007** | 0.1051 | 0.0835 | 0.1306 | 21.7947 | 21.3242 | 22.2652 |
| **2008** | 0.1132 | 0.0909 | 0.1393 | 21.6413 | 21.1783 | 22.1044 |
| **2009** | 0.0934 | 0.0735 | 0.1171 | 20.5839 | 20.1372 | 21.0306 |
| **2010** | 0.0822 | 0.0637 | 0.1044 | 21.6574 | 21.2029 | 22.112 |
| **2011** | 0.0846 | 0.0659 | 0.1068 | 21.2687 | 20.8244 | 21.713 |
| **2012** | 0.0917 | 0.0723 | 0.1148 | 19.673 | 19.2545 | 20.0916 |
| **2013** | 0.0915 | 0.0721 | 0.1145 | 18.7835 | 18.3818 | 19.1853 |
| **2014** | 0.0718 | 0.0548 | 0.0925 | 18.141 | 17.7528 | 18.5292 |
| **2015** | 0.0964 | 0.0765 | 0.1198 | 19.1956 | 18.8027 | 19.5886 |
| **2016** | 0.0961 | 0.0764 | 0.1195 | 20.0856 | 19.6898 | 20.4815 |
| **2017** | 0.1114 | 0.09 | 0.1363 | 20.0811 | 19.6917 | 20.4706 |
| **2018** | 0.1216 | 0.098 | 0.1452 | 20.2303 | 19.8453 | 20.6153 |
| **2019** | 0.1296 | 0.1052 | 0.1541 | 20.0302 | 19.653 | 20.4075 |
| **2020** | 0.1534 | 0.1268 | 0.1801 | 25.8375 | 25.4152 | 26.2598 |
| **2021** | 0.1605 | 0.1333 | 0.1877 | 14.1618 | 13.7664 | 14.5572 |
| **2022** | 0.1818 | 0.1527 | 0.2109 | 15.8437 | 15.4267 | 16.2608 |
| **2023** | 0.1348 | 0.1097 | 0.1599 | 16.6391 | 16.2135 | 17.0647 |

| **Supplementary Table 05: Alzheimer's Disease with Diabetes Mellitus-related Age-adjusted Mortality rate Stratified by Race among middle-aged and older adults in the United States, 1999-2023** | | | | | | | | | | | | | | | |
| --- | --- | --- | --- | --- | --- | --- | --- | --- | --- | --- | --- | --- | --- | --- | --- |
| **Year** | **American Indian or Alaska Native** | | | **Asian or Pacific Islander** | | | **Black or African American** | | | **White** | | | **Hispanic or Latino** | | |
|  | **AAMR** | **Lower 95%  CI** | **Upper 95%  CI** | **AAMR** | **Lower 95%  CI** | **Upper 95%  CI** | **AAMR** | **Lower 95%  CI** | **Upper 95%  CI** | **AAMR** | **Lower 95%  CI** | **Upper 95%  CI** | **AAMR** | **Lower 95%  CI** | **Upper 95%  CI** |
| **1999** | Unreliable | 1.7764 | 5.0469 | 1.9148 | 1.4503 | 2.4809 | 4.0119 | 3.6699 | 4.3538 | 2.7412 | 2.6579 | 2.8245 | 2.6923 | 2.2993 | 3.0853 |
| **2000** | Unreliable | 0.8068 | 3.0941 | 1.6353 | 1.2176 | 2.1501 | 4.2804 | 3.9294 | 4.6314 | 3.0763 | 2.9884 | 3.1643 | 3.1955 | 2.7774 | 3.6136 |
| **2001** | Unreliable | 1.2393 | 3.8035 | 1.8911 | 1.4656 | 2.4016 | 4.4083 | 4.053 | 4.7636 | 3.1868 | 3.098 | 3.2756 | 3.7863 | 3.3415 | 4.2312 |
| **2002** | Unreliable | 1.3026 | 3.9977 | 1.9316 | 1.5141 | 2.4287 | 5.1474 | 4.7651 | 5.5298 | 3.3981 | 3.3068 | 3.4894 | 3.8495 | 3.4103 | 4.2887 |
| **2003** | 3.9299 | 2.4912 | 5.8967 | 1.953 | 1.551 | 2.4274 | 5.494 | 5.1 | 5.8879 | 3.5587 | 3.4659 | 3.6515 | 4.2475 | 3.7994 | 4.6957 |
| **2004** | Unreliable | 1.5221 | 4.1835 | 2.1463 | 1.7344 | 2.6266 | 5.5594 | 5.1651 | 5.9538 | 3.6782 | 3.5844 | 3.7719 | 4.3043 | 3.8644 | 4.7441 |
| **2005** | 4.6027 | 3.0825 | 6.6103 | 2.7332 | 2.2569 | 3.2095 | 6.146 | 5.7368 | 6.5551 | 3.9466 | 3.8501 | 4.0432 | 4.6927 | 4.2485 | 5.1369 |
| **2006** | 3.5812 | 2.3176 | 5.2865 | 2.7251 | 2.264 | 3.1862 | 6.0123 | 5.6101 | 6.4146 | 3.9221 | 3.8267 | 4.0175 | 4.5081 | 4.0839 | 4.9322 |
| **2007** | 5.2107 | 3.6496 | 7.2139 | 3.1227 | 2.6452 | 3.6002 | 6.2469 | 5.842 | 6.6517 | 3.9522 | 3.8572 | 4.0472 | 4.7814 | 4.3577 | 5.2051 |
| **2008** | 3.9683 | 2.6369 | 5.7353 | 3.2937 | 2.8185 | 3.7688 | 5.9031 | 5.5149 | 6.2914 | 3.971 | 3.8764 | 4.0657 | 4.8999 | 4.4833 | 5.3164 |
| **2009** | 5.2921 | 3.745 | 7.2639 | 3.2555 | 2.799 | 3.7121 | 5.9262 | 5.5424 | 6.31 | 3.7312 | 3.64 | 3.8224 | 4.7651 | 4.3665 | 5.1637 |
| **2010** | 5.1087 | 3.6328 | 6.9837 | 3.8038 | 3.3199 | 4.2876 | 5.7832 | 5.4092 | 6.1571 | 3.9291 | 3.8361 | 4.0221 | 5.0566 | 4.6547 | 5.4585 |
| **2011** | 4.7397 | 3.3541 | 6.5056 | 3.8114 | 3.3504 | 4.2724 | 5.7465 | 5.3796 | 6.1135 | 3.8264 | 3.7354 | 3.9174 | 5.2685 | 4.875 | 5.6621 |
| **2012** | 4.2756 | 3.0104 | 5.8934 | 3.2144 | 2.807 | 3.6218 | 5.4308 | 5.0808 | 5.7807 | 3.6453 | 3.5569 | 3.7338 | 4.7697 | 4.4084 | 5.1309 |
| **2013** | 4.6233 | 3.3459 | 6.2275 | 2.9135 | 2.5407 | 3.2863 | 4.9416 | 4.6134 | 5.2699 | 3.4973 | 3.4116 | 3.583 | 5.0346 | 4.6744 | 5.3949 |
| **2014** | 4.4994 | 3.2819 | 6.0206 | 2.9126 | 2.5554 | 3.2698 | 4.9251 | 4.602 | 5.2482 | 3.3923 | 3.3082 | 3.4763 | 5.1256 | 4.7738 | 5.4773 |
| **2015** | 4.8077 | 3.5683 | 6.3383 | 3.303 | 2.937 | 3.6691 | 5.1091 | 4.7864 | 5.4318 | 3.6272 | 3.5408 | 3.7135 | 5.5437 | 5.1896 | 5.8977 |
| **2016** | 4.5184 | 3.3746 | 5.9253 | 3.2399 | 2.8877 | 3.5921 | 5.7919 | 5.4524 | 6.1315 | 3.8319 | 3.7441 | 3.9197 | 5.4302 | 5.0877 | 5.7728 |
| **2017** | 4.1059 | 3.0376 | 5.4283 | 3.2518 | 2.9123 | 3.5914 | 5.5312 | 5.2057 | 5.8566 | 3.9402 | 3.8518 | 4.0286 | 5.129 | 4.8064 | 5.4516 |
| **2018** | 3.9759 | 2.951 | 5.2417 | 3.4012 | 3.0625 | 3.7399 | 5.217 | 4.9074 | 5.5266 | 3.9718 | 3.8841 | 4.0595 | 5.7498 | 5.4162 | 6.0833 |
| **2019** | 3.2333 | 2.3303 | 4.3705 | 3.25 | 2.9291 | 3.571 | 5.2329 | 4.9277 | 5.5381 | 3.9738 | 3.8869 | 4.0607 | 5.7636 | 5.4361 | 6.091 |
| **2020** | 5.404 | 4.2716 | 6.7444 | 4.452 | 4.0878 | 4.8162 | 7.6725 | 7.3079 | 8.0372 | 4.9994 | 4.9025 | 5.0962 | 8.1235 | 7.7436 | 8.5035 |
| **2021** | 5.5781 | 4.5077 | 6.8262 | 4.1438 | 3.7865 | 4.5011 | 6.5658 | 6.2242 | 6.9074 | 5.0788 | 4.9822 | 5.1753 | 7.0777 | 6.7189 | 7.4365 |
| **2022** | 3.3833 | 2.5998 | 4.3287 | 4.0459 | 3.7107 | 4.381 | 5.7086 | 5.3999 | 6.0174 | 4.6551 | 4.5661 | 4.7441 | 6.7976 | 6.4578 | 7.1373 |
| **2023** | 3.3763 | 2.6166 | 4.2877 | 3.6451 | 3.3316 | 3.9587 | 5.4455 | 5.144 | 5.747 | 4.4009 | 4.3141 | 4.4878 | 5.7925 | 5.4806 | 6.1044 |

| **Supplementary Table 06: Alzheimer's Disease with Diabetes Mellitus-related Deaths Stratified by Region among middle-aged and older adults in the United States, 1999-2023** | | | | | | | | | | | | |
| --- | --- | --- | --- | --- | --- | --- | --- | --- | --- | --- | --- | --- |
| **Year** | **CENS-R1**  **Northeast** | | | **CENS-R2**  **Midwest** | | | **CENS-R3**  **South** | | | **CENS-R4**  **West** | | |
|  | **AAMR** | **Lower 95%  CI** | **Upper 95%  CI** | **AAMR** | **Lower 95%  CI** | **Upper 95%  CI** | **AAMR** | **Lower 95%  CI** | **Upper 95%  CI** | **AAMR** | **Lower 95%  CI** | **Upper 95%  CI** |
| **1999** | 2.4242 | 2.2677 | 2.5806 | 2.9552 | 2.7925 | 3.1178 | 3.0052 | 2.8671 | 3.1433 | 2.8004 | 2.6216 | 2.9792 |
| **2000** | 2.6548 | 2.4922 | 2.8173 | 3.3837 | 3.2102 | 3.5571 | 3.2926 | 3.1489 | 3.4364 | 3.1302 | 2.9436 | 3.3168 |
| **2001** | 2.6785 | 2.5166 | 2.8405 | 3.4474 | 3.2736 | 3.6211 | 3.4111 | 3.2658 | 3.5565 | 3.486 | 3.2917 | 3.6802 |
| **2002** | 2.7268 | 2.5644 | 2.8893 | 3.782 | 3.601 | 3.9631 | 3.7584 | 3.6067 | 3.9102 | 3.6573 | 3.46 | 3.8546 |
| **2003** | 2.6442 | 2.4851 | 2.8034 | 4.1437 | 3.9552 | 4.3321 | 4.0336 | 3.8773 | 4.1898 | 3.7784 | 3.5806 | 3.9763 |
| **2004** | 2.947 | 2.7801 | 3.1138 | 4.1785 | 3.99 | 4.367 | 4.0114 | 3.8566 | 4.1662 | 4.0467 | 3.8437 | 4.2497 |
| **2005** | 2.9698 | 2.8039 | 3.1358 | 4.5719 | 4.3762 | 4.7675 | 4.3224 | 4.1637 | 4.4811 | 4.4386 | 4.229 | 4.6482 |
| **2006** | 2.8464 | 2.6847 | 3.008 | 4.4038 | 4.2132 | 4.5944 | 4.2885 | 4.1322 | 4.4448 | 4.5988 | 4.3887 | 4.809 |
| **2007** | 2.9116 | 2.7488 | 3.0745 | 4.3826 | 4.1943 | 4.5709 | 4.5116 | 4.3531 | 4.6701 | 4.5332 | 4.3272 | 4.7392 |
| **2008** | 2.8016 | 2.6431 | 2.9602 | 4.4502 | 4.2613 | 4.639 | 4.3907 | 4.236 | 4.5454 | 4.7617 | 4.5528 | 4.9707 |
| **2009** | 2.5816 | 2.4297 | 2.7335 | 4.2822 | 4.0979 | 4.4666 | 4.2208 | 4.0706 | 4.3709 | 4.4993 | 4.2991 | 4.6995 |
| **2010** | 2.7221 | 2.5676 | 2.8766 | 4.3924 | 4.2065 | 4.5783 | 4.3462 | 4.1953 | 4.4971 | 4.9251 | 4.718 | 5.1322 |
| **2011** | 2.6144 | 2.4638 | 2.765 | 4.2953 | 4.113 | 4.4776 | 4.1371 | 3.9923 | 4.2819 | 5.091 | 4.8838 | 5.2981 |
| **2012** | 2.6147 | 2.4651 | 2.7644 | 4.1563 | 3.9779 | 4.3346 | 3.9036 | 3.7648 | 4.0423 | 4.5442 | 4.3509 | 4.7376 |
| **2013** | 2.4225 | 2.2793 | 2.5657 | 3.8672 | 3.6972 | 4.0373 | 3.7114 | 3.5781 | 3.8447 | 4.71 | 4.5155 | 4.9044 |
| **2014** | 2.254 | 2.1158 | 2.3923 | 3.9842 | 3.8113 | 4.1571 | 3.5971 | 3.4674 | 3.7267 | 4.5655 | 4.3777 | 4.7533 |
| **2015** | 2.3016 | 2.1626 | 2.4405 | 3.9498 | 3.7793 | 4.1203 | 4.0249 | 3.8899 | 4.1598 | 4.9982 | 4.8039 | 5.1925 |
| **2016** | 2.4731 | 2.3309 | 2.6154 | 4.3617 | 4.1841 | 4.5394 | 4.1929 | 4.0568 | 4.3289 | 5.0773 | 4.8846 | 5.2701 |
| **2017** | 2.3589 | 2.2215 | 2.4963 | 4.5306 | 4.3516 | 4.7096 | 4.2997 | 4.1637 | 4.4357 | 5.0402 | 4.8502 | 5.2302 |
| **2018** | 2.6223 | 2.479 | 2.7656 | 4.3841 | 4.2093 | 4.5589 | 4.4322 | 4.2965 | 4.568 | 5.0045 | 4.8184 | 5.1906 |
| **2019** | 2.5647 | 2.4239 | 2.7055 | 4.6022 | 4.4249 | 4.7796 | 4.3296 | 4.1972 | 4.462 | 4.9808 | 4.7978 | 5.1639 |
| **2020** | 3.3414 | 3.181 | 3.5017 | 5.8851 | 5.6852 | 6.0849 | 5.7067 | 5.5569 | 5.8565 | 6.5042 | 6.297 | 6.7113 |
| **2021** | 2.8982 | 2.7457 | 3.0506 | 5.2613 | 5.0659 | 5.4568 | 5.4746 | 5.3232 | 5.6261 | 6.4319 | 6.2191 | 6.6448 |
| **2022** | 2.6052 | 2.4652 | 2.7452 | 4.7347 | 4.5559 | 4.9135 | 5.0541 | 4.9145 | 5.1938 | 5.7818 | 5.5887 | 5.975 |
| **2023** | 2.5535 | 2.4144 | 2.6926 | 4.6162 | 4.4382 | 4.7942 | 4.7796 | 4.6439 | 4.9153 | 5.2686 | 5.0837 | 5.4536 |

| **Supplementary Table 07: Alzheimer's Disease with Diabetes Mellitus-related Deaths Stratified by Urbanization among middle-aged and older adults in the United States, 1999-2020** | | | | | | |
| --- | --- | --- | --- | --- | --- | --- |
| **Year** | **Metropolitan** | | | **Non-metropolitan** | | |
|  | **AAMR** | **Lower 95%  CI** | **Upper 95%  CI** | **AAMR** | **Lower 95%  CI** | **Upper 95%  CI** |
| **1999** | 2.7085 | 2.6226 | 2.7944 | 3.3072 | 3.1146 | 3.4997 |
| **2000** | 3.0034 | 2.9135 | 3.0932 | 3.7381 | 3.5342 | 3.9421 |
| **2001** | 3.1299 | 3.0391 | 3.2206 | 3.874 | 3.6673 | 4.0807 |
| **2002** | 3.3383 | 3.2451 | 3.4315 | 4.3224 | 4.1044 | 4.5404 |
| **2003** | 3.5111 | 3.4165 | 3.6057 | 4.5212 | 4.2992 | 4.7431 |
| **2004** | 3.5967 | 3.5016 | 3.6917 | 4.799 | 4.5705 | 5.0276 |
| **2005** | 3.9033 | 3.8053 | 4.0014 | 5.1089 | 4.8743 | 5.3435 |
| **2006** | 3.8449 | 3.7486 | 3.9411 | 5.1193 | 4.8866 | 5.3521 |
| **2007** | 3.8737 | 3.7782 | 3.9693 | 5.3905 | 5.153 | 5.628 |
| **2008** | 3.899 | 3.8041 | 3.9939 | 5.255 | 5.0215 | 5.4885 |
| **2009** | 3.7079 | 3.6161 | 3.7996 | 5.0636 | 4.8354 | 5.2918 |
| **2010** | 3.8801 | 3.787 | 3.9732 | 5.4056 | 5.1711 | 5.6402 |
| **2011** | 3.8256 | 3.7345 | 3.9167 | 5.1784 | 4.9505 | 5.4063 |
| **2012** | 3.6258 | 3.5379 | 3.7137 | 4.9014 | 4.681 | 5.1217 |
| **2013** | 3.5238 | 3.4382 | 3.6095 | 4.5959 | 4.3849 | 4.8069 |
| **2014** | 3.4617 | 3.3777 | 3.5456 | 4.4338 | 4.2274 | 4.6401 |
| **2015** | 3.7283 | 3.6423 | 3.8144 | 4.6459 | 4.4357 | 4.8561 |
| **2016** | 3.8802 | 3.7936 | 3.9669 | 5.1 | 4.8819 | 5.3181 |
| **2017** | 3.8735 | 3.7879 | 3.9591 | 5.4483 | 5.2247 | 5.672 |
| **2018** | 3.9876 | 3.9021 | 4.0731 | 5.2029 | 4.9868 | 5.419 |
| **2019** | 3.933 | 3.8491 | 4.017 | 5.5156 | 5.2947 | 5.7365 |
| **2020** | 5.1793 | 5.084 | 5.2747 | 6.9329 | 6.6867 | 7.179 |

| **Supplementary Table 08 : Alzheimer's Disease-related Deaths Stratified by Sex among middle-aged and older adults in the United States, 1999-2023** | | | |
| --- | --- | --- | --- |
| **Year** | **Overall** | **Male** | **Female** |
| **1999** | 78686 | 25105 | 53581 |
| **2000** | 84174 | 26100 | 58074 |
| **2001** | 88635 | 27372 | 61263 |
| **2002** | 95955 | 29450 | 66505 |
| **2003** | 99446 | 30589 | 68857 |
| **2004** | 101166 | 31169 | 69997 |
| **2005** | 107524 | 32958 | 74566 |
| **2006** | 106923 | 33001 | 73922 |
| **2007** | 108069 | 33335 | 74734 |
| **2008** | 111027 | 34416 | 76611 |
| **2009** | 105851 | 33285 | 72566 |
| **2010** | 109982 | 34818 | 75164 |
| **2011** | 109850 | 34513 | 75337 |
| **2012** | 107011 | 34025 | 72986 |
| **2013** | 107470 | 34042 | 73428 |
| **2014** | 115764 | 36359 | 79405 |
| **2015** | 134601 | 42407 | 92194 |
| **2016** | 141563 | 44801 | 96762 |
| **2017** | 146891 | 46780 | 100111 |
| **2018** | 147683 | 47515 | 100168 |
| **2019** | 146659 | 47353 | 99306 |
| **2020** | 174145 | 56037 | 118108 |
| **2021** | 151049 | 48983 | 102066 |
| **2022** | 150005 | 48838 | 101167 |
| **2023** | 140077 | 45956 | 94121 |
| **Total** | 2,970,206 | 939,207 | 2030999 |

| **Supplemental Table 09: Overall and Sex-stratified Alzheimer's Disease-related Age-Adjusted Mortality Rate among middle-aged and older adults in the United States, 1999-2023** | | | | | | | | | |
| --- | --- | --- | --- | --- | --- | --- | --- | --- | --- |
| **Year** | **Male** | | | **Female** | | | **Overall** | | |
|  | **AAMR** | **Lower 95%  CI** | **Upper 95%  CI** | **AAMR** | **Lower 95%  CI** | **Upper 95%  CI** | **AAMR** | **Lower 95%  CI** | **Upper 95%  CI** |
| **1999** | 41.7267 | 41.2015 | 42.2519 | 46.238 | 45.8452 | 46.6308 | 45.0114 | 44.6969 | 45.326 |
| **2000** | 42.7389 | 42.212 | 43.2658 | 49.4283 | 49.0248 | 49.8318 | 47.3862 | 47.066 | 47.7064 |
| **2001** | 43.8848 | 43.357 | 44.4125 | 51.4453 | 51.0363 | 51.8542 | 49.0977 | 48.7744 | 49.4209 |
| **2002** | 46.4361 | 45.898 | 46.9741 | 55.2776 | 54.8559 | 55.6994 | 52.4735 | 52.1414 | 52.8055 |
| **2003** | 47.1287 | 46.5937 | 47.6636 | 56.4417 | 56.0184 | 56.8651 | 53.4568 | 53.1245 | 53.7891 |
| **2004** | 47.0789 | 46.5501 | 47.6076 | 56.8351 | 56.4121 | 57.258 | 53.6241 | 53.2936 | 53.9546 |
| **2005** | 48.2721 | 47.746 | 48.7983 | 59.3566 | 58.9283 | 59.785 | 55.7299 | 55.3967 | 56.0632 |
| **2006** | 46.9521 | 46.4418 | 47.4623 | 57.7035 | 57.2847 | 58.1224 | 54.0947 | 53.7701 | 54.4192 |
| **2007** | 46.0523 | 45.5552 | 46.5493 | 57.2468 | 56.8328 | 57.6608 | 53.433 | 53.114 | 53.7521 |
| **2008** | 46.2308 | 45.7402 | 46.7215 | 57.7419 | 57.3289 | 58.1549 | 53.7748 | 53.4578 | 54.0919 |
| **2009** | 43.5952 | 43.1252 | 44.0653 | 53.6591 | 53.2639 | 54.0543 | 50.1354 | 49.8323 | 50.4385 |
| **2010** | 44.631 | 44.1606 | 45.1013 | 54.6956 | 54.2992 | 55.092 | 51.1814 | 50.8776 | 51.4852 |
| **2011** | 42.4272 | 41.9782 | 42.8762 | 53.3602 | 52.9729 | 53.7475 | 49.4951 | 49.2007 | 49.7896 |
| **2012** | 40.6035 | 40.1705 | 41.0364 | 50.7712 | 50.3964 | 51.1459 | 47.1151 | 46.8308 | 47.3993 |
| **2013** | 39.2842 | 38.8653 | 39.7032 | 50.0848 | 49.7159 | 50.4537 | 46.1839 | 45.9057 | 46.4622 |
| **2014** | 40.7256 | 40.3052 | 41.1459 | 53.3525 | 52.9747 | 53.7303 | 48.7015 | 48.4187 | 48.9843 |
| **2015** | 46.1588 | 45.7174 | 46.6001 | 60.8281 | 60.4284 | 61.2277 | 55.4133 | 55.1149 | 55.7118 |
| **2016** | 47.5309 | 47.0886 | 47.9731 | 62.8511 | 62.4483 | 63.2538 | 57.1644 | 56.8643 | 57.4645 |
| **2017** | 48.166 | 47.7273 | 48.6046 | 64.1066 | 63.7035 | 64.5097 | 58.1267 | 57.8274 | 58.4261 |
| **2018** | 47.4387 | 47.0101 | 47.8673 | 63.0365 | 62.6413 | 63.4318 | 57.1316 | 56.8387 | 57.4246 |
| **2019** | 45.9912 | 45.575 | 46.4074 | 61.7762 | 61.3881 | 62.1644 | 55.7451 | 55.4585 | 56.0316 |
| **2020** | 53.1356 | 52.6934 | 53.5779 | 72.6796 | 72.2614 | 73.0978 | 65.1395 | 64.8324 | 65.4467 |
| **2021** | 49.2269 | 48.7865 | 49.6672 | 67.8533 | 67.4349 | 68.2717 | 60.5509 | 60.2445 | 60.8572 |
| **2022** | 46.3868 | 45.9715 | 46.8021 | 61.7469 | 61.3644 | 62.1295 | 55.8003 | 55.5173 | 56.0833 |
| **2023** | 42.7628 | 42.3672 | 43.1584 | 59.2001 | 58.8212 | 59.579 | 52.7204 | 52.4436 | 52.9971 |

| **Supplemental Table 10: Annual percent change (APC) Alzheimer's Disease -related Age-Adjusted Mortality Rate among middle-aged and older adults in the United States, 1999-2023** | | | | | | | |
| --- | --- | --- | --- | --- | --- | --- | --- |
| **Year Interval** | **APC** | | **Lower 95%  CI** | **Upper 95%  CI** | | **P-value** | |
| **Overall** | | | | | | | |
| **1999-2005** | 3.4965* | 1.0876 | | | 5.9627 | | 0.007379 |
| **2005-2012** | -2.4556* | -4.5290 | | | -0.3373 | | 0.026365 |
| **2012-2020** | 3.6103* | 1.9968 | | | 5.2493 | | 0.000259 |
| **2020-2023** | -5.6171* | -10.6572 | | | -0.2926 | | 0.040340 |
| **1999-2023** | 0.5954 | -0.5181 | | | 1.7213 | | 0.295901 |
| **Male** | | | | | | | |
| **1999-2005** | 2.3939* | 0.3439 | | | 4.4857 | | 0.025035 |
| **2005-2013** | -2.1713* | -3.5736 | | | -0.7486 | | 0.005686 |
| **2013-2020** | 3.5085* | 1.8388 | | | 5.2055 | | 0.000456 |
| **2020-2023** | -5.8865* | -10.1004 | | | -1.4750 | | 0.013092 |
| **1999-2023** | 0.1071 | -0.8215 | | | 1.0445 | | 0.821836 |
| **Female** | | | | | | | |
| **1999-2005** | 4.0034* | 1.5114 | | | 6.5566 | | 0.003742 |
| **2005-2012** | -2.2651* | -4.4122 | | | -0.0698 | | 0.044079 |
| **2012-2020** | 4.0255* | 2.3423 | | | 5.7363 | | 0.000137 |
| **2020-2023** | -5.5614* | -10.7867 | | | -0.0302 | | 0.048952 |
| **1999-2023** | 0.9175 | -0.2384 | | | 2.0869 | | 0.120208 |

| **Supplementary Table 11: Diabetes Mellitus-related Deaths Stratified by Sex among middle-aged and older adults in the United States, 1999-2023** | | | |
| --- | --- | --- | --- |
| **Year** | **Overall** | **Male** | **Female** |
| **1999** | 209354 | 98727 | 110627 |
| **2000** | 212751 | 100846 | 111905 |
| **2001** | 217790 | 103778 | 114012 |
| **2002** | 223760 | 108170 | 115590 |
| **2003** | 227228 | 111071 | 116157 |
| **2004** | 225122 | 110856 | 114266 |
| **2005** | 233232 | 115697 | 117535 |
| **2006** | 230690 | 115385 | 115305 |
| **2007** | 231046 | 116373 | 114673 |
| **2008** | 233439 | 118213 | 115226 |
| **2009** | 230307 | 118319 | 111988 |
| **2010** | 233725 | 120730 | 112995 |
| **2011** | 238872 | 123992 | 114880 |
| **2012** | 240729 | 126410 | 114319 |
| **2013** | 246432 | 130626 | 115806 |
| **2014** | 244673 | 131373 | 113300 |
| **2015** | 252454 | 136254 | 116200 |
| **2016** | 258473 | 140980 | 117493 |
| **2017** | 270272 | 148365 | 121907 |
| **2018** | 275500 | 152921 | 122579 |
| **2019** | 282372 | 157577 | 124795 |
| **2020** | 387461 | 217695 | 169766 |
| **2021** | 398708 | 225009 | 173699 |
| **2022** | 368857 | 207288 | 161569 |
| **2023** | 338877 | 191795 | 147082 |
| **Total** | 6,512,124 | 83,117 | 3,083,674 |

| **Supplemental Table 12: Overall and Sex-stratified Diabetes Mellitus-related Age-Adjusted Mortality Rate among middle-aged and older adults in the United States, 1999-2023** | | | | | | | | | |
| --- | --- | --- | --- | --- | --- | --- | --- | --- | --- |
| **Year** | **Male** | | | **Female** | | | **Overall** | | |
|  | **AAMR** | **Lower 95%  CI** | **Upper 95%  CI** | **AAMR** | **Lower 95%  CI** | **Upper 95%  CI** | **AAMR** | **Lower 95%  CI** | **Upper 95%  CI** |
| **1999** | 137.7157 | 136.8405 | 138.591 | 104.8812 | 104.2598 | 105.5025 | 118.467 | 117.9595 | 118.9745 |
| **2000** | 138.8625 | 137.99 | 139.7351 | 104.8659 | 104.2481 | 105.4838 | 118.9875 | 118.4818 | 119.4931 |
| **2001** | 139.812 | 138.9463 | 140.6777 | 105.3877 | 104.7726 | 106.0029 | 119.8088 | 119.3055 | 120.312 |
| **2002** | 142.8858 | 142.0187 | 143.753 | 105.4835 | 104.8721 | 106.0949 | 121.1161 | 120.614 | 121.6182 |
| **2003** | 143.3595 | 142.5014 | 144.2175 | 104.6092 | 104.0042 | 105.2142 | 120.9395 | 120.4418 | 121.4372 |
| **2004** | 140.4036 | 139.5623 | 141.2449 | 101.6471 | 101.0543 | 102.24 | 117.9892 | 117.5011 | 118.4773 |
| **2005** | 143.0905 | 142.2516 | 143.9294 | 102.8596 | 102.2676 | 103.4516 | 119.8979 | 119.4103 | 120.3855 |
| **2006** | 139.2778 | 138.4607 | 140.0949 | 99.2345 | 98.6572 | 99.8118 | 116.3177 | 115.8416 | 116.7938 |
| **2007** | 137.1775 | 136.3766 | 137.9785 | 97.0337 | 96.467 | 97.6004 | 114.2421 | 113.7745 | 114.7097 |
| **2008** | 136.0262 | 135.238 | 136.8143 | 95.6914 | 95.1335 | 96.2494 | 113.0366 | 112.5758 | 113.4973 |
| **2009** | 132.6924 | 131.9235 | 133.4614 | 91.504 | 90.962 | 92.0461 | 109.3314 | 108.882 | 109.7808 |
| **2010** | 133.034 | 132.2704 | 133.7976 | 90.9697 | 90.4325 | 91.507 | 109.1267 | 108.681 | 109.5725 |
| **2011** | 132.2274 | 131.4785 | 132.9762 | 90.382 | 89.8514 | 90.9127 | 108.5771 | 108.1376 | 109.0165 |
| **2012** | 130.9729 | 130.2377 | 131.708 | 87.9512 | 87.4329 | 88.4694 | 106.7401 | 106.3092 | 107.171 |
| **2013** | 131.415 | 130.6891 | 132.1409 | 87.4381 | 86.9256 | 87.9506 | 106.7483 | 106.3218 | 107.1747 |
| **2014** | 128.3815 | 127.674 | 129.0891 | 83.9752 | 83.4771 | 84.4733 | 103.5176 | 103.1022 | 103.9331 |
| **2015** | 129.6548 | 128.9527 | 130.3569 | 84.3875 | 83.8929 | 84.8821 | 104.3925 | 103.9796 | 104.8054 |
| **2016** | 130.9668 | 130.2693 | 131.6643 | 83.8 | 83.3113 | 84.2888 | 104.6456 | 104.2362 | 105.055 |
| **2017** | 134.1698 | 133.4734 | 134.8661 | 85.1464 | 84.6589 | 85.6338 | 106.878 | 106.4692 | 107.2869 |
| **2018** | 134.8352 | 134.1467 | 135.5237 | 84.0639 | 83.5842 | 84.5436 | 106.6038 | 106.2 | 107.0075 |
| **2019** | 135.7156 | 135.0331 | 136.3981 | 83.8953 | 83.4211 | 84.3696 | 106.9983 | 106.598 | 107.3985 |
| **2020** | 183.6794 | 182.8932 | 184.4656 | 112.6707 | 112.1241 | 113.2174 | 144.4792 | 144.0173 | 144.9411 |
| **2021** | 190.7361 | 189.9273 | 191.545 | 119.2606 | 118.6894 | 119.8319 | 151.3951 | 150.9171 | 151.8732 |
| **2022** | 171.301 | 170.5479 | 172.0541 | 105.3824 | 104.8589 | 105.9058 | 134.6461 | 134.2055 | 135.0867 |
| **2023** | 155.7342 | 155.0221 | 156.4464 | 95.6133 | 95.1172 | 96.1095 | 122.6135 | 122.1949 | 123.032 |

| **Supplemental Table 13: Annual percent change (APC) of Diabetes Mellitus-related Age-Adjusted Mortality Rate among middle-aged and older adults in the United States, 1999-2023** | | | | | | | |
| --- | --- | --- | --- | --- | --- | --- | --- |
| **Year Interval** | **APC** | | **Lower 95%  CI** | **Upper 95%  CI** | | **P-value** | |
| **Overall** | | | | | | | |
| **1999-2018** | -0.9213* | -1.0956 | | | -0.7467 | | < 0.000001 |
| **2018-2021** | 14.4764* | 8.7973 | | | 20.4520 | | 0.000031 |
| **2021-2023** | -11.1482* | -15.3876 | | | -6.6963 | | 0.000089 |
| **1999-2023** | 0.0277 | -0.7385 | | | 0.6881 | | 0.939237 |
| **Male** | | | | | | | |
| **1999-2018** | -0.4863* | -0.6670 | | | -0.3053 | | 0.000028 |
| **2018-2021** | 14.6272* | 9.0741 | | | 20.4631 | | 0.000021 |
| **2021-2023** | -10.9754* | -15.1219 | | | 6.6263 | | 0.000081 |
| **1999-2023** | 0.3523 | -0.3453 | | | 1.0549 | | 0.323114 |
| **Female** | | | | | | | |
| **1999-2018** | -1.5221* | -1.6966 | | | -1.3473 | | < 0.000001 |
| **2018-2021** | 14.0300* | 8.0374 | | | 20.3550 | | 0.000083 |
| **2021-2023** | -11.2939* | -15.7929 | | | -6.5545 | | 0.000148 |
| **1999-2023** | -0.5702 | -1.3195 | | | 0.1849 | | 0.138517 |
